# Engulfing cells promote neuronal regeneration and remove neuronal debris through distinct biochemical functions of CED-1

**DOI:** 10.1038/s41467-018-07291-x

**Published:** 2018-11-19

**Authors:** Hui Chiu, Yan Zou, Nobuko Suzuki, Yi-Wen Hsieh, Chiou-Fen Chuang, Yi-Chun Wu, Chieh Chang

**Affiliations:** 1 Department of Biological Sciences, University of Illinois at Chicago, Chicago, Illinois 60607 USA; 2 Institute of Molecular and Cellular Biology, National Taiwan University, Taipei, 10617 Taiwan; 3 Institute of Atomic and Molecular Sciences, Academia Sinica, Taipei, 10617 Taiwan; 4 Present Address: Division of Biology and Biological Engineering and HHMI, California Institute of Technology, Pasadena, CA 91125 USA; 5 Present Address: School of Life Science, ShanghaiTech University, Shanghai, 200031 China

## Abstract

Two important biological events happen coincidently soon after nerve injury in the peripheral nervous system in *C. elegans*: removal of axon debris and initiation of axon regeneration. But, it is not known how these two events are co-regulated. Mutants of *ced-1*, a homolog of Draper and MEGF10, display defects in both events. One model is that those events could be related. But our data suggest that they are actually separable. CED-1 functions in the muscle-type engulfing cells in both events and is enriched in muscle protrusions in close contact with axon debris and regenerating axons. Its two functions occur through distinct biochemical mechanisms; extracellular domain-mediated adhesion for regeneration and extracellular domain binding-induced intracellular domain signaling for debris removal. These studies identify CED-1 in engulfing cells as a receptor in debris removal but as an adhesion molecule in neuronal regeneration, and have important implications for understanding neural circuit repair after injury.

## Abstract (web summary)

It is unclear how removal of axon debris and initiation of axon regeneration following nerve injury is co-regulated. In this study, the authors show that the extracellular domain (ECD) of the engulfment receptor, CED-1, functions as an adhesion molecule to promote axonal regeneration after injury, independent of its function in phagocytosis

## Introduction

Rapid removal of axon debris after neuronal trauma is essential for the injured neuron to regenerate effectively^1–3^. Failure to remove axon debris could damage neurons by triggering the inflammatory immune responses. Also, remnant axon debris may become physical barrier to hinder axon regeneration^4^. Therefore, efficient removal of axon debris helps neurons recover from trauma and re-establish neural connections. Previous studies on axon regeneration have identified many intrinsic and extrinsic molecules that either block or promote regeneration. However, it is unclear how axon regeneration is mechanistically related to clearance of axon debris after neuronal injury.

Axon debris arising from neuronal injury, like cell corpses arising from apoptosis, is removed by either professional or amateur engulfing cells. Professional engulfing cells, such as macrophages, exhibit high motility^5^. They are capable of identifying damaged tissues or dying cells at a distance and removing debris through rapid internalization and degradation. In contrast, amateur engulfing cells are less motile and their internalization of cell debris is slow^6^. Amateur engulfing cells are usually neighboring cells, responding to local cues released from injured neurons or dying cells. *C. elegans* does not have professional phagocytes, thus the apoptotic or necrotic cell corpses are removed by amateur engulfing cells, including hypodermal cells, body wall muscles, gonadal sheath cells, and intestinal cells^7,8^.

Upon receiving the eat-me signal from dying cells or axon debris, engulfing cells initiate the removal process. The eat-me signal is recognized by specialized receptors expressed on engulfing cells. The gene *ced-1* (ced stands for cell death abnormal) encodes a transmembrane scavenger receptor that is highly conserved between invertebrates and vertebrates^7^. CED-1 and its homologues, including Draper in fly and MEGF-10 and Jedi in mammals, are the major engulfment receptors that function in engulfing cells for cell corpse removal^9–17^. In addition, Draper has been recently shown to mediate glial clearance of degenerating axon debris caused by either axon pruning or neuronal trauma^18,19^. These observations suggest a central role for CED-1 during evolution in removing cell corpses and axon debris.

**Fig. 1:**
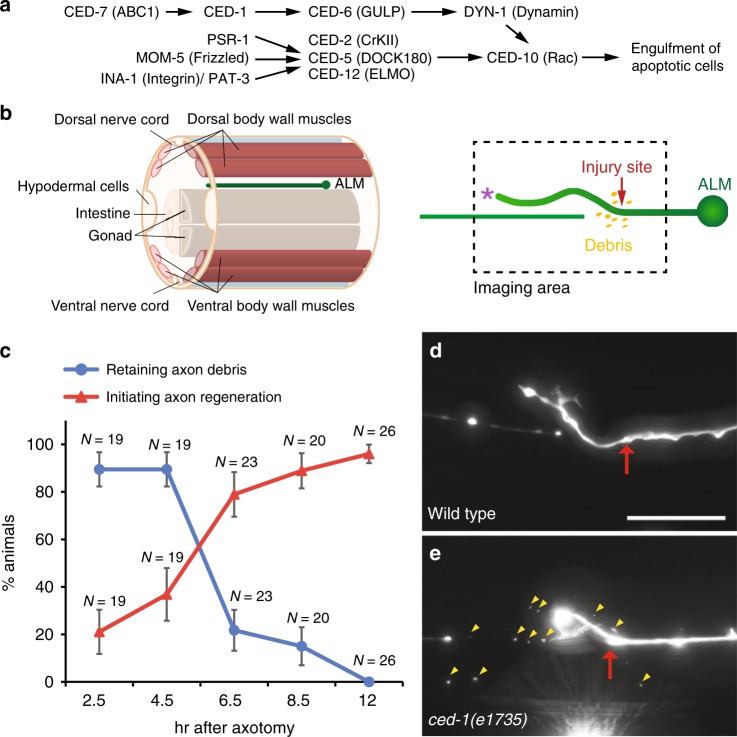
Axon debris removal is tightly linked with axon regeneration initiation. **a** Two genetic pathways work redundantly or in parallel to remove apoptotic cells in *C. elegans*. **b** Schematic of a cross-sectioned animal. The ALM axon and various engulfing cells were shown. A lateral view of regenerated ALM axon and resulting axon debris was shown following laser axotomy. **c** The percentage of animals retaining axon debris surrounding the lesion site or initiating axon regeneration was determined at various time points following laser surgery. The percentage of animals retaining axon debris decreased from 89.47 to 0% (Blue circles) while the percentage of animals initiating axon regeneration increased from 21.05 to 96% (Red triangles) within 12 h following laser axotomy. The N number represents the number of animals analyzed. Error bars represent SEP. **d** By 12 h after laser surgery, axon debris was removed completely in wild-type animals carrying the axonal marker, *zdIs4[Pmec-4::GFP]*. **e** Axon debris remained surrounding the lesion site in *ced-1(e1735)* mutants 12 h after laser surgery. Dorsal is up; anterior is to the left in all images. Red arrows indicate lesion sites and yellow arrowheads point to axon debris. Scale bar: 20 μm

The recognition and engulfment of cell corpses in nematode requires at least two redundant signaling pathways^20^ (Fig. 1a). One involves the transthyretin-like TTR-52, the engulfment receptor CED-1, the adaptor protein CED-6 (GULP), and the ABC transporter CED-7 (ABCA)^7,21–26^. TTR-52 acts as a bridging factor that mediates recognition of cell corpses by bridging the phosphatidylserine (PtdSer) eat-me signal with the engulfment receptor CED-1^21^. CED-1 activates engulfing cells through the adaptor proteins CED-6 and CED-7^22,24^. CED-6 transmits the eat-me signal from CED-1 to DYN-1 (dynamin), a downstream component required for internalization and degradation of cell corpses^24,25^. CED-7 functions in both dying cells and engulfing cells^22^. It has been suggested that CED-7 helps present “eat-me” signals on the surface of cell corpses and cluster CED-1 receptors on the membrane of engulfing cells^7,22,27^. In addition, CED-7 may facilitate adhesion between these two cells by transporting adhesion-related molecules to the cell surface^26^. The other involves INA-1/PAT-3, PSR-1 (phosphatidylserine receptor), MOM-5 (Frizzled), CED-2 (CrkII), CED-5 (DOCK180), CED-12 (ELMO), and CED-10 (Rac GTPase)^28–37^. INA-1/PAT-3, PSR-1, and MOM-5 receptors transduce the “eat-me” signal through CED-2^34-36^. As a canonical component, CED-2 recruits CED-5 and CED-12 proteins to the cell membrane of engulfing cells, where CED-5 and CED-12 function together as a guanine nucleotide exchange factor to facilitate the exchange of GDP for GTP on CED-10, leading to cytoskeleton rearrangement and engulfment of dying cells^28–33,37^.

Fragments of injured axons that detach from their cell bodies break down by the molecularly regulated process of Wallerian degeneration^38,39^. It has been proposed that delayed removal of axon debris broken down from these fragments in CNS blocks regeneration in the axon that remains connected to the cell body^40,41^. Here, we show that after axotomy, proximal debris is removed and axons regenerate. Both processes are affected in *ced-1* mutants. One possibility is that those processes could be related (e.g., axon debris removal facilitates axon regeneration). But our data indicate that they are actually separable. CED-1 functions in engulfing cells in both processes and its two functions are mediated through separable biochemical pathways (extracellular domain-mediated adhesion for regeneration and extracellular domain binding-induced intracellular domain signaling for debris removal). Other engulfment genes are also involved in axon regeneration. *ced-5* can function both cell-autonomously in touch neurons and non-cell-autonomously in three types of engulfing cells to promote axon regeneration. *ced-6* (GULP) inhibits axon regeneration through negative regulation of CED-1.

CED-1, Draper, and MEGF10 (SR-F3) homologues have been studied predominantly as receptors for cell engulfment. But a recent study showed that MEGF10 (SR-F3) also mediates cell–cell repulsion^42^. Here, we report a novel and unexpected role of CED-1 in neuronal regeneration. We show that the CED-1 protein functions in the muscle-type of engulfing cells not only for axon debris removal but also for axon regeneration. The ectodomain (ECD) of CED-1 acts as an adhesion molecule from the engulfing cell surface to promote axon regeneration in neurons.

## Results

### Axon debris removal is linked to axon regeneration

*C. elegans* has been utilized as a genetic model to identify novel cellular and molecular mechanisms underlying nervous system regeneration^43–47^. Time-lapse imaging of axon debris occurrence and axon regeneration following laser axotomy of the ALM touch neuron (Fig. 1b) showed that axon debris disappearance coincides with axon regeneration initiation between 4.5 and 6.5 h after injury (Fig. 1c), suggesting that axon debris disappearance is tightly linked to axon regeneration initiation. By 12 h after laser surgery, axon debris was removed completely in wild-type animals (Fig. 1d), whereas axon debris remained surrounding the lesion site in *ced-1(e1735)* mutants (Fig. 1e).

### CED-1 acts in muscles for debris removal and axon regrowth

**Fig. 2:**
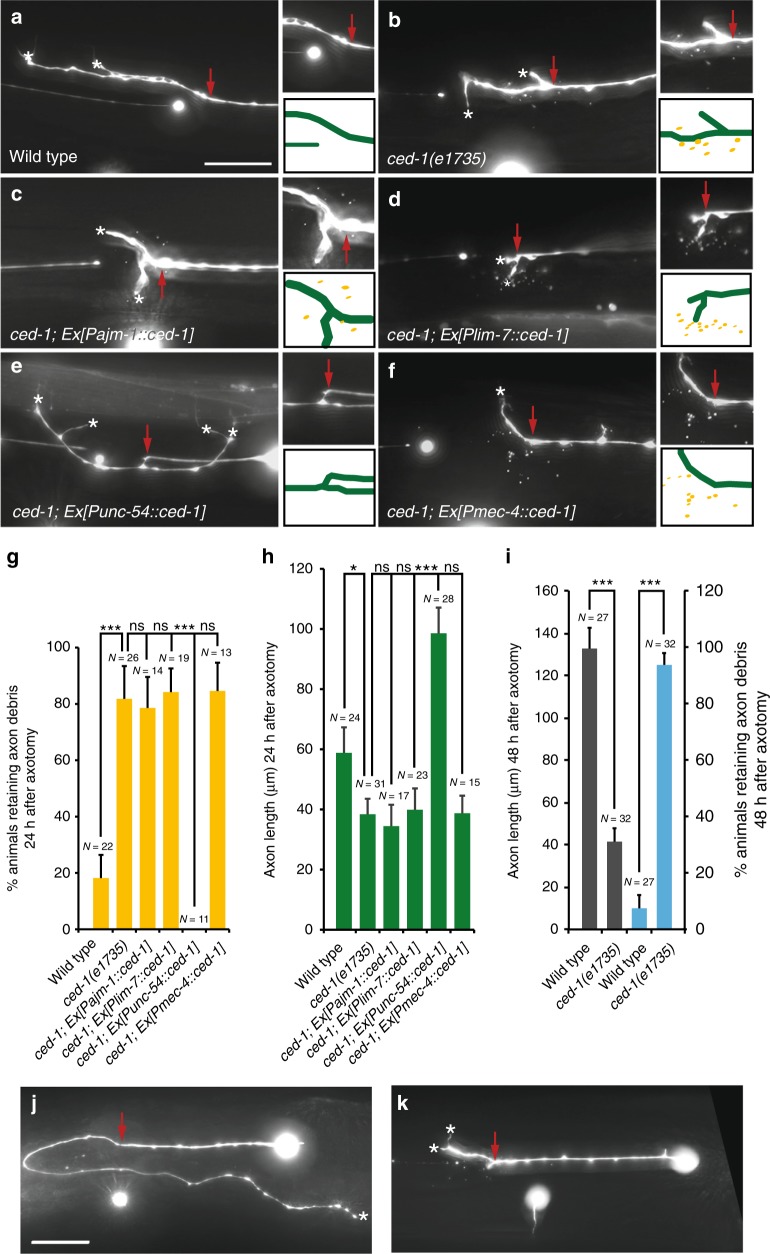
c*ed-1* acts specifically in the muscle-type engulfing cells to remove axon debris and promote axon regeneration. **a**–**f** All images were taken 24 h after laser axotomy. Axon trajectories and debris were visualized using the *zdIs4[Pmec-4::GFP]* marker. Dorsal is up; anterior is to the left. Red arrows indicate lesion sites. Scale bar: 20 μm. **a** Wild-type animals removed axon debris completely and regrew axons well. **b** *ced-1(e1735)* mutants showed accumulation of axon debris around the proximal end of the injured axon and reduced axon regeneration. **c**–**f** Rescue experiments using cell-specific promoters, the *Pajm-1*, the *Plim-7*, the *Punc-54*, and the *Pmec-4* to re-express *ced-1* in hypodermal cells, gonadal sheath cells, muscles, and touch neurons, respectively, in *ced-1* mutants. **c** Re-expressing *ced-1* in hypodermal cells failed to rescue the accumulation of axon debris and the reduced axon regeneration in *ced-1* mutants. **d** Axon debris remained around the lesion site and axon regeneration was still limited in *ced-1* mutants carrying the *Plim-7::ced-1* transgene. **e** Axon debris was entirely removed and robust axon regeneration was observed in *ced-1* mutants carrying the *Punc-54::ced-1* transgene. **f** Axon debris accumulated and axon regeneration limited in *ced-1* mutants carrying the *Pmec-4::ced-1* transgene. **g** Quantification of the percentages of animals retaining axon debris 24 h after laser axotomy. Error bars represent SEP. ***p < 0.001 by *Z*-test for two proportions. **h** Average length of regenerating ALM axons 24 h after laser axotomy. Error bars indicate SEM. * and *** indicate *p* < 0.05 and 0.001, respectively. *P* values were calculated using a Student’s *t*-Test. **i** Quantification of average length of ALM axon regeneration and the percentages of animals retaining axon debris 48 h after laser axotomy. The N number represents the number of animals analyzed. ns indicates no significant difference. Error bars indicate SEM for axon length and SEP for % animals. **j** Wild-type animals removed axon debris around the proximal end of the injured axon and regrew much longer axons 48 h after axotomy. **k** *ced-1(e1735)* mutants still showed accumulation of axon debris and reduced axon regeneration 48 h after axotomy. Scale bar: 20 μm

**Fig. 3:**
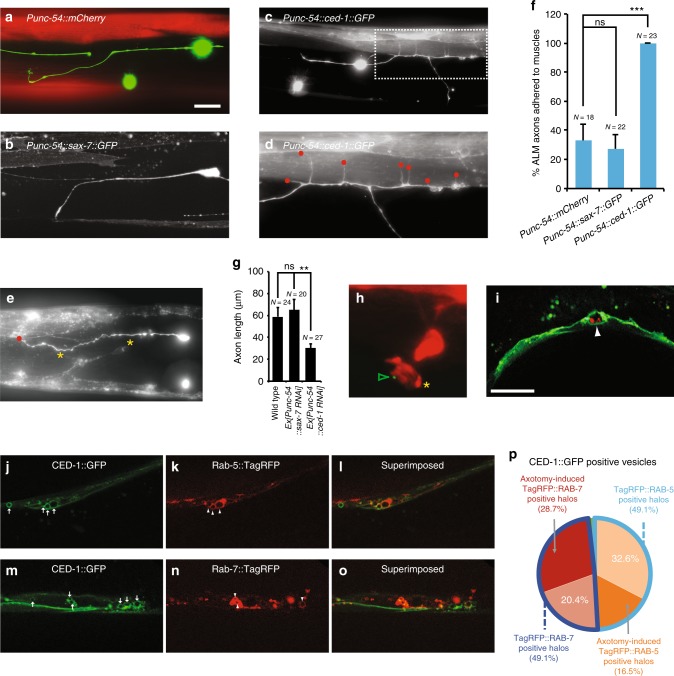
CED-1 from the muscle-type engulfing cell is adhesive to regenerating axons and mediates axon debris phagocytosis. **a**–**f** Regenerating ALM axons were adhered to muscles overexpressing CED-1. Representative images were taken in young adult stage 24 h after injury (**a–e**). Anterior is to the left; dorsal is up. Scale bar: 20 μm. **a** The ALM axon in wild-type animals expressing the *Punc-54::mCherry* transgene was not adhered to muscles. **b** The ALM axon in wild-type animals expressing the *Punc-54::sax-7* transgene was not adhered to muscles. **c**–**e** ALM axons in wild-type animals expressing the *Punc-54::ced-1* transgene were adhered to muscles. The dashed box area in (**c**) was blown up in (**d**). Red dots in (**d**) and (**e**) indicate regenerating axon endings at muscles and yellow asterisks in (**e**) mark muscle protrusions. **f** The percentages of regenerating ALM axons adhered to muscles. The N number represents the number of animals analyzed. Error bars represent SEP. Asterisks represent *P* < 0.001 by *Z*-test for two proportions. **g** Average length of regenerating ALM axons. The muscle-specific knockdown of *ced-1* caused reduced axon regeneration. Axons were visualized using the *zdIs4[Pmec-4::GFP]* marker. ns indicates no significant difference. Error bars indicate SEM. **p < 0.01. *P* values were calculated using a Student’s *t*-Test. **h** Axon debris were labeled by the *Pmec-4::GFP* transgene and muscle protrusions were labeled by the *Punc-54::ced-1::mRFP* reporter. The green open arrowhead and the asterisk point to axon debris and the muscle protrusion, respectively. **i** Axon debris were labeled by the *Pmec-4::myr::mCherry* transgene and body wall muscles were labeled by the *Punc-54::ced-1::GFP* reporter. The white arrowhead points to a phagosome encircling axon debris. Images were taken in young adult stage 4 h after axotomy for axon debris (**h** and **i**). Scale bar: 10 μm. **j** and **m** CED-1::GFP-expressing vesicles in muscle cells. Early phagosomes (**k**) and late phagosomes (**n**) in muscle cells were labeled by the Rab-5::TagRFP and the Rab-7::TagRFP, respectively. **l** and **o** Superimposed images. Arrows indicate CED-1::GFP-expressing vesicles whereas arrowheads mark either Rab-5::TagRFP-positive halos (**k**) or Rab-7::TagRFP-positive halos (**n**). **p** The percentages of CED-1::GFP positive vesicles that are also positive for either Rab-5::TagRFP or Rab-7::TagRFP

These results suggest two models. One model is that removal of axon debris is a prerequisite for axon regeneration. An alternative model is that engulfing cells that are required for axon debris removal may also be used for axon regeneration. Here, our study supports the latter model. *ced-1* mutations caused significant accumulation of axon debris and significantly reduced axon regeneration 24 h following ALM axotomy (Fig. 2a, b, g, h). The analysis of axon debris clearance and axon regeneration in *ced-1* mutants 48 h after axotomy still showed significant deficiencies, albeit more severe in 48 h than 24 h following axotomy (Fig. 2i, j, k). These results indicate that *ced-1* mutations caused defects rather than a simple delay in axon debris clearance and axon regeneration. *ced-1* is normally expressed in three types of engulfing cells at the adult stage (Supplementary Figure 1). Cell-specific rescue experiments showed that CED-1 is required specifically in the muscle type of engulfing cells to remove axon debris near the proximal segment of injured axons (Fig. 2b-g). In addition, cell-specific rescue experiments showed that CED-1 also specifically functions in the muscle type of engulfing cells to promote axon regeneration (Fig. 2b-f, h). RNAi of *ced-1* but not another cell adhesion molecule *sax-7* (L1CAM) in the muscle type of engulfing cells significantly reduced ALM axon regeneration (Fig. 3g).

### CED-1 functions as an adhesion protein in muscles for axons

When *ced-1* transmembrane proteins were over-expressed in body wall muscles, we found that these muscles had a tendency to adhere ALM axons during both axon regeneration following injury (Fig. 3a–f) and initial axon outgrowth in development (Supplementary Figure 2a-e). In many cases, we observed axon regeneration in action coupled with muscle protrusions in the vicinity (Fig. 3e; yellow asterisks). These muscle protrusions are in a good position to support axon regeneration and/or to guide axon regeneration. In addition, regenerating axons can grow from either the proximal end of the severed axon or the cell body away from the lesion site (Fig. 3c, e). 100% of regenerating ALM axons were adhered to muscles overexpressing *ced-1* as opposed to only 33% of regenerating ALM axons were adhered to wild-type muscles and 27% of regenerating ALM axons were adhered to muscles overexpressing another cell adhesion molecule *sax-7* (L1CAM) (P < 0.001; Fig. 3f). These results suggest that CED-1 may function as an adhesion protein from muscles to keep regenerating axons in close association with muscles, which likely allows other growth-promoting factors to increase regeneration. Similar observations were made during initial ALM axon outgrowth in development (Supplementary Figure 2). Ninety percent of developing ALM axons were adhered to muscles overexpressing *ced-1* as opposed to only 28% of developing ALM axons were adhered to wild-type muscles and 28% of developing ALM axons were adhered to muscles overexpressing *sax-7* (L1CAM) (P < 0.001; Supplementary Figure 2e). Analysis of ALM axon trajectory in *ced-1* mutants frequently discovered a curved (52% of *ced-1(e1735)* ALM axons contain at least one sharp turn, *n* = 22; Supplementary Figure 2g, h) instead of a wt-like straight axon (Supplementary Figure 2a), suggesting that ALM axon pathfinding may be affected in *ced-1* mutants.

**Fig. 4:**
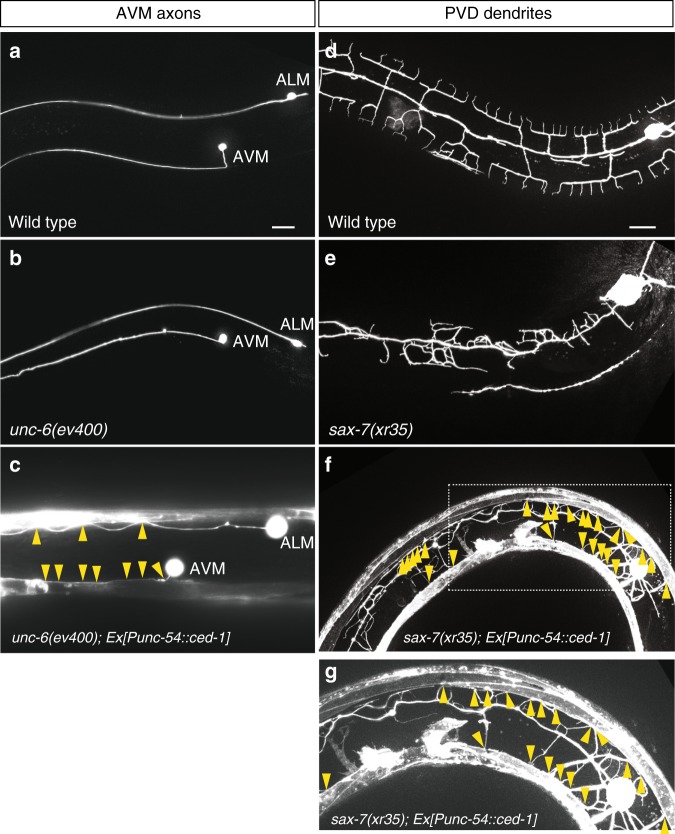
CED-1-overexpressing muscle-type engulfing cells adhere AVM axons and PVD dendrites. Representative images showing wild-type AVM axons (**a**), *unc-6* mutant AVM axons (**b**), wild-type PVD dendrites (**d**), and *sax-7* mutant PVD dendrites (**e**). AVM axons (**c**) and PVD dendrites (**f**) without their respective guidance cues, *unc-6* (netrin) and *sax-7* (L1CAM), were redirected and adhered to muscles expressing CED-1. AVM neurons were labeled by *zdIs5[Pmec-4::GFP]* and PVD neurons were labeled by *xrIs37[PF49H12.4::GFP]*. The dashed box area in (**f**) was blown up and shown in (**g**). Arrowheads in (**c**), (**f**), and (**g**) indicate muscle contacts. Anterior is to the left, dorsal is up. Scale bar, 20 μm

To further support CED-1’s role as an adhesion molecule, we tested the *ced-1*’s effect from the muscle-type engulfing cells on other neurites, AVM axons and PVD dendrites. In wild-type animals, AVM axons are guided to ventral nerve cord due to combined actions of dorsal repulsion from SLT-1 (slit) cue and ventral attraction to UNC-6 (netrin) cue produced by the ventral nerve cord motor neurons (Fig. 4a). In *unc-6* mutants, AVM axons instead project anteriorly and adopt a lateral position (Fig. 4b). Expression of CED-1 in body wall muscles redirected AVM axons in *unc-6* mutants to muscles (Fig. 4c; % AVM axons redirected to muscles = 100%, *n* > 30). In wild-type animals, PVD dendrites are guided to skin cells (Fig. 4d) due to combined effects of two skin cues, SAX-7 (L1CAM) and MNR-1, and one muscle cue, LECT-2. In *sax-7* mutants, PVD dendrites fail to extend to skin cells (Fig. 4e). Expression of CED-1 in body wall muscles redirected PVD dendrites in *sax-7* mutants to muscles (Figs. 4f, g; % PVD dendrites redirected to muscles = 100%, *n* > 30). Together, these results further support a role of CED-1 in muscles as an adhesion molecule to neurites.

Using a 5-kb *ced-1* promoter mCherry reporter to label engulfing cells and a 1-kb *mec-4* promoter GFP marker to label regenerating ALM axons, we were able to observe muscles extending a protrusion that appears to be in contact with regenerating axons 12 h following axotomy (Supplementary Figure 3a-c). In an alternative approach, we used a muscle-specific promoter driven *ced-1::mRFP* reporter to simultaneously label muscle cells and monitor the CED-1 protein distribution. With this specific reporter, the protrusion could be seen to extend from muscles to the proximity of regenerating axons (Supplementary Figure 3d-i) and axon debris (Fig. 3h), further suggesting a role for the muscle-type engulfing cells in axon regeneration and axon debris removal. Enrichment of the CED-1::mRFP fusion protein in muscles can be seen in protrusions (Supplementary Figure 3e, h) and in regions contacting with regenerating axons (Supplementary Figure 3e, f), consistent with CED-1 being instructive in the process. We used time-lapse microscopy to monitor morphological changes in axons after surgery and found that transient filopodia appeared at approximately the same time and the same frequency in *ced-1* mutants as in wild-type animals (Supplementary Figure 4). However, axon regeneration after injury was significantly reduced in *ced-1* mutants compared to wild-type animals (Fig. 2h, i). This result suggests that, during axon regeneration, *ced-1* is required to transform exploratory filopodia into growth cones rather than the initial step of filopodial extension.

### *ced-1*-mediated phagocytosis is involved in debris removal

RAB-5 is a well-established early endosome marker, and RAB-7 labels late endosomes and lysosomes. In addition, it has been previously shown that the small GTPases RAB-5 and RAB-7 are required for maturation of apoptotic-cell-containing phagosomes^48^. RAB-5 preferentially localizes to early phagosomes containing uncondensed cell corpses, whereas RAB-7 preferentially localizes to late phagosomes containing highly refractile apoptotic-cell corpses^48^. We found that, before axotomy, 32.6% of the *ced-1*-expressing vesicles are labeled by the Rab-5 marker (Fig. 3p), whereas 20.4% of the *ced-1*-expressing vesicles are labeled by the Rab-7 marker inside the muscle cell (Fig. 3p). These vesicles could represent early endosomes (CED-1 and Rab-5 positive) or late endosomes and lysosomes (CED-1 and Rab-7 positive) that are native to muscle cells. Alternatively, they could represent non-axotomy-induced phagosomes that carry apoptotic cells or other cargos. In contrast, during axon debris removal following axotomy, 49.1% of the *ced-1*-expressing vesicles are labeled by the Rab-5 marker (Fig. 3j-l, p) whereas 49.1% of the *ced-1*-expressing vesicles are labeled by the Rab-7 marker inside the muscle cell (Fig. 3m–p). These results suggest that about 16.5% of the *ced-1*-expressing vesicles are axotomy-induced early phagosomes and about 28.7% of the *ced-1*-expressing vesicles are axotomy-induced late phagosomes. In addition, we observed a *ced-1*-expressing muscle protrusion approaches axon debris (Fig. 3h) and axon debris inside a *ced-1*-expressing phagosome (Fig. 3i). Together, these results indicate that *ced-1*-mediated phagocytosis may be involved in axon debris removal.

### Roles of p38 & JNK pathways in CED-1-mediated axon regrowth

A recent study showed that axon regeneration requires the coordinate activation of p38 and JNK MAPK pathways in *C. elegans*^49,50^. In addition, Draper (CED-1 homolog) has been shown to act through JNK to mediate proper engulfment of dying germline cells *in Drosophila*^51^. To ask whether the activation of JNK and p38 pathways is essential for CED-1-mediated axon regeneration and axon debris clearance after injury, we tested the potential of the mutations in either the JNK or the p38 pathway to block the ability of the *Punc-54::ced-1* transgene to rescue the *ced-1* mutant phenotype of reducing axon regeneration and accumulating axon debris. RNAi of the JNK pathway components (*mlk-1* and *mek-1*) blocked CED-1-mediated axon regeneration and axon debris clearance, suggesting that both CED-1-mediated axon regeneration and axon debris clearance require the activation of the JNK pathway (Supplementary Figure 5a, b). The same RNAi feeding bacterial clones for knocking down *mlk-1* and *mek-1* genes also caused reduced ALM axon regeneration in wild-type animals. RNAi of the p38 pathway components (*dlk-1 and pmk-3*), while blocking CED-1-mediated axon regeneration, had less effect on CED-1-mediated axon debris clearance (Supplementary Figure 5a, b). These results suggest that the p38 pathway is more critical for CED-1-mediated axon regeneration than axon debris removal.

### The ECD of CED-1 acts as an adhesion molecule for axons

**Fig. 5:**
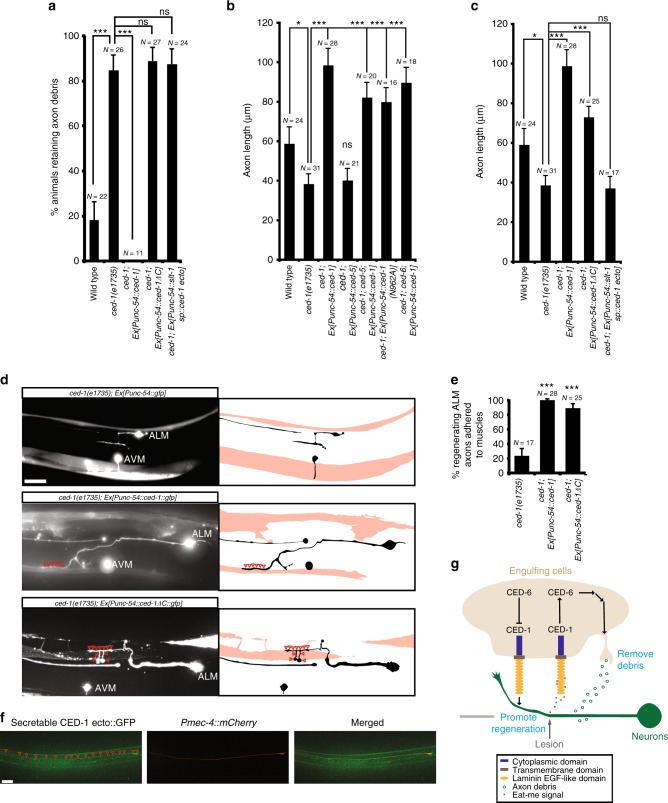
The ectodomain of CED-1 can function from the muscle-type engulfing cell surface to promote axon regeneration. **a** The percentages of animals retaining axon debris 24 h after axotomy. Error bars represent SEP. ***p < 0.001 by *Z*-test for two proportions. **b** Average length of regenerating ALM axons. **c** Average length of regenerating ALM axons in *ced-1* mutants expressing various transgenes 24 h after axotomy. *Punc-54::ced-1* transgenes expressed either the full-length CED-1 protein (bar 3), the cytoplasmic domain-deleted CED-1 protein (bar 4), or the ectodomain only CED-1 protein (bar 5). In **b** and **c**, error bars indicate SEM; * and *** indicate *p* < 0.05 and *p* < 0.001, respectively; *P* values were calculated using a Student’s *t*-Test. **d** Representative images showing adhesion of regenerating ALM axons to muscles re-expressing either the wild-type CED-1 protein or the cytoplasmic domain-deleted CED-1 protein in *ced-1* mutants. Regenerating ALM axon in *ced-1* mutants alone did not adhere to body wall muscles. Images were taken in young adult stage 24 h after injury. Anterior is to the left; dorsal is up. Scale bar: 20 μm. Open red arrowheads mark axon contact regions. The axon and muscle tracing of each image was shown to the right. **e** The percentages of regenerating ALM axons adhered to muscles 24 h after injury. Error bars represent SEP. Asterisks represent *P* < 0.001 by *Z*-test for two proportions. **f** The CED-1 ectodomain-GFP fusion proteins were bound to the ALM process and soma. Single focal-plane images of the secretable CED-1 ectodomain-GFP fusion protein distribution in the extracellular environment (left), the ALM mCherry marker expression (middle), and the superimposed (right) were shown. Arrowheads indicate the enrichment of CED-1 ectodomain-GFP fusion proteins in the ALM axon and the cell body. Scale bar: 20 μm. **g** Model of distinct biochemical functions of CED-1. Injury signals released from damaged axons attract engulfing cells, which leads to removal of axon debris and promotion of axon regeneration. CED-1 functions in engulfing cells in both events through distinct biochemical pathways

To understand further molecular mechanisms by which the CED-1 transmembrane protein promotes axon regeneration and axon debris clearance, we deleted the entire cytoplasmic domain of the CED-1 protein and tested its ability to rescue the *ced-1* mutant phenotype of accumulating axon debris and reducing axon regeneration. We found that, like the previous report by others^52^, the CED-1 protein lacking the cytoplasmic domain was no longer able to remove axon debris (Fig. 5a). However, to our surprise, it remained able to promote axon regeneration from body wall muscles (*ced-1* versus *ced-1; Ex[Punc-54::ced-1 ΔC]*, *P* < 0.001; Fig. 5c), to a similar extent as the CED-1(N962A) mutant protein (*ced-1; Ex[Punc-54::ced-1 ΔC]* versus *ced-1; Ex[Punc-54::ced-1(N962A)*, *P* = 0.445; Fig. 5b, c). It was previously shown that the NPXY motif (residues 962–965) in the CED-1 cytoplasmic domain mediates the interaction with the PTB domain in CED-6^7^ and that the CED-1(N962A) mutant protein, which loses the ability to bind to CED-6, loses 85% of its native activity to remove cell corpses^7^. We also noticed that the cytoplasmic domain-deleted CED-1 protein did not promote axon regeneration as well as the wild-type CED-1 protein (*ced-1; Ex[Punc-54::ced-1 ΔC]* versus *ced-1; Ex[Punc-54::ced-1]*, *P* = 0.0175), likely because the truncated protein is less stable. Furthermore, the secretable CED-1 ectodomain protein, which lacks the transmembrane and the cytoplasmic domains of the protein, lost the ability to promote ALM axon regeneration (Fig. 5c). The secretable CED-1 ectodomain protein was expressed from the body wall muscle and was made secretable using a *slt-1* signal peptide (Fig. 5c). Taken together, these results suggest that the ectodomain of CED-1 functions from the engulfing cell surface to promote axon regeneration in neurons. Separately, the cytoplasmic domain of CED-1 is required for axon debris removal (Fig. 5g).

Further analysis of ALM axons in *ced-1* mutants re-expressing the *Punc-54::ced-1 ΔC* transgene showed that when the truncated CED-1 protein lacking the cytoplasmic domain was re-expressed in the muscle-type engulfing cells in *ced-1* mutants, it can still adhere ALM axons during axon regeneration following injury (Fig. 5d, e). The extent of axon adhesion during regeneration is similar to that displayed by the wild-type CED-1 protein re-expressed in the muscle-type engulfing cells in *ced-1* mutants (Fig. 5d, e). These results support a model in which CED-1 functions in muscles as an adhesion molecule to keep the regenerating axon attached to the muscle, which likely allows other growth-promoting factors to increase regeneration (Fig. 5g). Further supporting this model, the CED-1 ectodomain protein, which lacks the transmembrane and the cytoplasmic domains of the protein and is secreted from the body wall muscle to the surrounding environment, was bound to the ALM axon and cell body (Fig. 5f).

### Axon regeneration involves selective engulfment genes

To understand the extent to which engulfment genes are utilized for axon regeneration, we performed a comprehensive analysis of mutant effects of engulfment genes on axon regeneration. Additional engulfment mutants that are known to affect clearance of cell corpses following cell death, including *ced-2*, *ced-5, ced-6, ced-7, ced-10*, *ced-12*, and *psr-1* mutants, were analyzed (Supplementary Figure 6). We found that, 24 h following ALM axotomy, *ced-2*, *ced-5*, *ced-10*, and *ced-12* mutants showed significantly reduced axon regeneration (Supplementary Figure 6c,d,h). These results suggest that they may act together to promote axon regeneration.

### *ced-5* acts in neurons and engulfing cells for axon regrowth

To identify cells that utilize the *ced-5*’s function to promote ALM axon regeneration, we analyzed the expression pattern of a 1.3-kb *ced-5* promoter GFP reporter in adult animals. Because of the complex genomic structure in the upstream regulatory region of the *ced-5* gene, we were unable to make a longer version of the *ced-5* promoter reporter. Thus, the expression pattern revealed by this reporter may not be comprehensive. This reporter was expressed in three types of engulfing cells, including intestinal cells, body wall muscles, and gonadal sheath cells (Supplementary Figure 7a-c). Even though the *ced-5* expression was not detected in hypodermal cells, its function in hypodermal cells was well documented during clearance of cell corpses. Similar to a recent report by others^53^, we found that *ced-5* can function cell-autonomously in neurons to promote axon regeneration. Our cell-specific rescue experiments using the transgene expressing *ced-5* from the touch neuron-specific *mec-4* promoter cell-autonomously rescued the *ced-5* mutant phenotype of reducing axon regeneration (Supplementary Figure 8f, g). To our surprise, the transgene expressing *ced-5* from the *ajm-1* (hypodermal cell specific), the *unc-54* (muscle cell specific), or the *lim-7* (gonadal sheath cell specific) promoter also rescued the *ced-5* mutant phenotype of reducing axon regeneration (Supplementary Figure 8a-e, g), which suggests a non-cell-autonomous role for *ced-5* in engulfing cells for axon regeneration. Cell-type-specific RNAi that preferentially silenced the expression of the CED-5 protein in either touch neurons (Supplementary Figure 9a, b) or muscle cells (Supplementary Figure 9a, c) significantly reduced ALM axon regeneration (Supplementary Figure 9d). Together, our results showed that *ced-5* can function both cell-autonomously in touch neurons and non-cell-autonomously in three types of engulfing cells to promote ALM axon regeneration.

### ced-6 inhibits axon regrowth through downregulating CED-1

Interestingly, our comprehensive analysis of mutant effects of engulfment genes on axon regeneration revealed that *ced-6* and *ced-7* mutants enhanced ALM axon regeneration (Supplementary Figure 6f, g, i), which suggests that engulfing cells in these two mutants may have greater ability to promote ALM axon regeneration than their wild-type counterparts. The enhanced ALM axon regeneration caused by *ced-6* mutations appears to be *ced-1* dependent, since this phenotype was suppressed by *ced-1* mutations (Supplementary Figure 10a). In addition, *ced-6* mutations significantly increased the frequency of CED-1::mRFP fusion proteins enriched on the muscle surface contacting with regenerating axons (Supplementary Figure 10b, c). It is not known how *ced-6* regulates *ced-1* muscle surface expression, but CED-6 and CED-1 proteins are co-localized to the surface area in muscles (Supplementary Figure 10d). Together, these results suggest that *ced-6* inhibits axon regeneration through downregulating CED-1 proteins on the surface of muscle-type engulfing cells. *ced-6* and *ced-7* mutants, like *ced-1* mutants, also displayed accumulation of axon debris 24 h after injury (Supplementary Figure 10e-h and Table 1). But, unlike *ced-1* mutants, their axon regeneration was not deterred (Supplementary Figure 6i). Thus, clearance of axon debris is not a prerequisite for axon regeneration. It was also previously shown in mice that peripheral nerve regeneration is not hindered by the retention of the distal axons^39^. In contrast to *ced-1*, *ced-6*, or *ced-7* mutants, *ced-5* mutants are normal in axon debris removal [Table 1; % animals retaining axon debris in *ced-5* (19.05%) versus wild type (17.39%), *P* = 0.9416]. Therefore, distinct sets of engulfment genes are selected to function in axon debris removal and axon regeneration.

**Table 1:** Genetic determinants of axon debris clearance

Strain^a^	*N*	Animals with axon debris	%	Animals without axon debris	%
*zdIs4*	22	4	18.18%	18	81.82%
*ced-1(e1735); zdIs4*	26	22	84.62%	4	15.38%
*ced-1; zdIs4; Ex[Pmec-4::ced-1]*	13	11	84.62%	2	15.38%
*ced-1; zdIs4; Ex[Pajm-1::ced-1]*	14	11	78.57%	3	21.43%
*ced-1; zdIs4; Ex[Plim-7::ced-1]*	19	16	84.21%	3	15.79%
*ced-1; zdIs4; Ex[Punc-54::ced-1]*	11	0	0.00%	11	100.00%
*ced-1; zdIs4; Ex[Punc-54::ced-1(N962A)]*	21	21	100.00%	0	0.00%
*ced-1; zdIs4; Ex[Punc-54::ced-1 deltaC]*	27	24	88.89%	3	11.11%
*ced-1; zdIs4; Ex[Punc-54::slt-1-sp::ced-1 ecto]*	24	21	87.50%	3	12.50%
*ced-1; zdIs4; Ex[Punc-54::ced-5]*	20	16	80.00%	4	20.00%
*ced-6(tm1826); zdIs4*	22	21	95.45%	1	4.55%
*ced-6; zdIs4; Ex[Punc-54::ced-6]*	23	11	47.83%	12	52.17%
*ced-6; zdIs4; Ex[Punc-54::ced-1]*	23	20	86.96%	3	13.04%
*ced-7(n1892); zdIs4*	26	25	96.15%	1	3.85%
*zdIs5*	23	4	17.39%	19	82.61%
*ced-5(n1812); zdIs5*	21	4	19.05%	17	80.95%
*ced-5; zdIs5; Ex[Pmec-4::ced-5]*	21	3	14.29%	18	85.71%
*ced-5; zdIs5; Ex[Pajm-1::ced-5]*	20	4	20.00%	16	80.00%
*ced-5; zdIs5; Ex[Plim-7::ced-5]*	24	5	20.83%	19	79.17%
*ced-5; zdIs5; Ex[Punc-54::ced-5]*	16	3	18.75%	13	81.25%
*psr-1(tm469); zdIs5*	22	5	22.73%	17	77.27%
*ced-10(n1993); zdIs5*	22	4	18.18%	18	81.82%
*ced-12(k149) zdIs5*	24	4	16.67%	20	83.33%
*ced-2(n1994); zdIs5*	22	4	18.18%	18	81.82%

^a^All strain names are in italic format

### *ced-1* promotes axon regrowth independent of *ced-5* and *ced-6*

To determine whether the CED-1 transmembrane protein requires the *ced-5*’s function in muscles to promote axon regeneration in neurons, we tested the potential of the *ced-5* null mutation *n1812* to block the ability of the *Punc-54::ced-1* transgene to rescue the *ced-1* mutant phenotype of reducing axon regeneration. *ced-5* mutations failed to block the ability of the *Punc-54::ced-1* transgene to rescue the *ced-1* mutant phenotype of reducing axon regeneration, suggesting that the *ced-1*’s function in muscles for axon regeneration does not require *ced-5* (Fig. 5b). Further, the *Punc-54::ced-5* transgene, which rescued the *ced-5* mutant phenotype of reducing axon regeneration (Supplementary Figure 8g), failed to rescue the *ced-1* mutant phenotype of reducing axon regeneration (Fig. 5b), which suggests that *ced-5* cannot bypass the requirement of *ced-1* for axon regeneration.

It was previously shown that CED-1 requires CED-6 in cell corpse removal^7^. Here, we find that *N962A* mutations do not abolish the ability of the *ced-1* transgene to promote axon regeneration, which indicates that *ced-1* does not require *ced-6* to promote axon regeneration (Fig. 5b). Opposite phenotypic effects in axon regeneration displayed by *ced-6* and *ced-1* mutants also suggest that *ced-6* is unlikely to mediate the *ced-1*’s function in axon regeneration. We nevertheless tested this possibility and find that, indeed, *ced-6* mutations do not block the ability of the *Punc-54::ced-1* transgene to rescue the *ced-1* mutant phenotype of reducing axon regeneration (Fig. 5b). Taken together, our results show that the *ced-1*’s function in muscle cells to promote axon regeneration in neurons does not require *ced-5* or *ced-6*.

## Discussion

Many extrinsic and intrinsic factors that act to promote or block axon regeneration after injury have been identified, but little is known for the mechanisms underlying removal of axon debris and its relationship to axon regeneration. One model is that those two events could be related. But our data suggest that clearance of axon debris is not a prerequisite for axon regeneration, so these two events are separable. We show that the engulfing cells that are required for axon debris removal are also used for axon regeneration. CED-1 functions in engulfing cells in both events through two distinct biochemical pathways: extracellular domain-mediated adhesion for regeneration and extracellular domain binding-induced intracellular domain signaling for debris removal. Engulfing cells are equipped to sense and respond to axon injury and thus are conveniently positioned to execute these two events together.

The transgene that we use to express *ced-1* in the hypodermal cell (*Pajm-1::ced-1*) is functional in rescuing the *ced-1* mutant phenotype in cell corpse removal^54^. However, the same transgene fails to rescue the *ced-1* mutant phenotype in axon regeneration or debris removal (Fig. 2g, h). Interestingly, the transgene that we used to express *ced-1* in the muscle cell (*Punc-54::ced-1*), while successfully rescuing the defects in axon regeneration and debris removal caused by the *ced-1* mutation (Fig. 2g, h), it fails to rescue the *ced-1* mutant phenotype in cell corpse removal^54^. Collectively, these results suggest that different engulfing cells use *ced-1* to play different roles in *C. elegans*. We show here a specific role of *ced-1* in the muscle-type engulfing cells for axon debris removal and regeneration.

A recent study discovered an interesting role for MEGF10 (SR-F3) in synapse elimination^55^. It was shown that astrocytes actively engulf central nervous system synapses through the MEGF10 (SR-F3) pathway^55^. Here, we report a novel and unexpected role of CED-1 in neuronal regeneration. We show that CED-1 functions in the muscle type of engulfing cells for axon debris removal and for axon regeneration.

Other engulfment genes are also involved in axon regeneration. *ced-5* (Dock180) acts in both engulfing cells and neurons to promote axon regeneration. Our results indicate that at least three types of engulfing cells are capable of promoting ALM axon regeneration in a *ced-5*-dependent manner. It is possible that various non-professional engulfing cells are broadly involved in axon regeneration using a *ced-5*-dependent mechanism, such as actin-cytoskeleton rearrangement that is required for not only mobilization of engulfing cells but also migration of axonal growth cones in regenerating neurons. However, these engulfing cells, except for the muscle cells, promote axon regeneration in a *ced-1*-independent manner.

ALM axon regeneration is enhanced in *ced-6* and *ced-7* mutants compared to that in wild-type animals (Supplementary Figure 6i). In some cellular context, while *ced-6* and *ced-7* mutations affecting the apoptotic-cell removal, they did not affect the ability of engulfing cells to sense the eat-me signal and to approach the cell corpse^7^. In this study, we also find that, at least in *ced-6* mutants, engulfing cells are capable of moving into close proximity to axon debris but unable to remove it. The persistent exposure to the eat-me signal released from un-removed axon debris in *ced-6* mutants would increase the frequency of interactions between engulfing cells and injured axons, which could contribute to enhancement of axon regeneration.

What is the molecular mechanism underlying the increase in regeneration in the *ced-6* mutant? Our genetic and imaging analysis demonstrate that *ced-6* (GULP) inhibits axon regeneration through downregulating CED-1 proteins on the surface of engulfing cells. One might imagine that CED-1 acts as an adhesion molecule and its cell surface expression in muscles is inhibited by CED-6 through a CED-1 ICD dependent or independent mechanism. Thus, in the *ced-6* mutant, more CED-1 adhesion molecules are present on the surface of the muscle protrusion, which can adhere the regenerating axon better and promote its regrowth.

To determine whether axonal regeneration in the presence of axon debris restores normal function in touch neurons, we examine anatomical connectivity of the regenerated ALM axon 24 h after axotomy in *ced-6* and *ced-7* mutants. We find that in *ced-6* and *ced-7* mutants, 75 and 81% (*n* = 24 and *n* = 26), respectively, of regenerated ALM axons fail to reconnect to or fuse with the distal disconnected axon segment. These results suggest that this axon regeneration in the presence of axon debris unlikely restores functional neuronal circuits in the majority of cases.

In newly hatched wild-type larvae, the ALM soma and axon lie next to the body wall muscle; as the animals mature, the ALM soma and axon are moved away from the muscle by becoming embedded in hypodermis^56^. In this study, we find that the CED-1-mediated adhesion allows the ALM soma and axon to remain attached to the muscle even as development progresses to adult (Supplementary Figure 2c,d). In some cases, we can also observe the CED-1-mediated adhesion stabilizes more than one growth cone from the AVM neuron in early development to form multiple muscle-adhered AVM axons (Supplementary Figure 2f). Hypodermis that is also damaged during axotomy would open up another opportunity to allow regenerating ALM axons to adhere to muscles through the CED-1-mediated adhesion.

Engulfment genes were also investigated for their roles in axonal fusion, a spontaneous event for regenerating axon to fuse with disconnected axon fragment. Inactivation of *ced-1* did not cause a significant defect on axon fusion, whereas animals lacking PSR-1, NRF-5, CED-6, or CED-7 displayed significant fusion defects^57^. Since there is only a small portion of regenerating ALM axons undergoing axon fusion (25%, *n* > 50), it is unclear what the relationship might be between axon fusion and axon regeneration in ALM neurons.

Both SCARF1 (SR-F1) and MEGF10 (SR-F3) have been recognized as mammalian orthologues of *C. elegans* CED-1^55,58^. The role of mammalian SCARFs in clearance of cell debris/corpses appears to be highly conserved in evolution. A recent report showed that SCARF1 (SR-F1), similar to CED-1, mediates cell corpse engulfment^59^. Another member of the SCARF family, MEGF10 (SR-F3), has recently been shown to be a receptor for C1q, an eat-me signal for apoptotic cells, and is required for phagocytosis of apoptotic neurons by astrocytes in the developing mouse brain in cerebellum^60^. Mammalian SCARFs, similar to CED-1, are also involved in cell–cell adhesion. SCARF2 (SR-F2), like SCARF1 (SR-F1), contains multiple EGF-like repeats in its extracellular domain^61^. However, unlike SCARF1 (SR-F1), SCARF2 (SR-F2) has little activity to internalize modified LDL^61^. Remarkably, in mouse fibroblast cells, intense cell–cell adhesion was observed only when SCARF1 (SR-F1)-expressing cells were mixed with SCARF2 (SR-F2)-expressing cells^61^. This heterophilic trans-interaction is mediated through the extracellular EGF-like repeats and independent of the cytoplasmic domain^61^. The association of SCARF1 (SR-F1) and SCARF2 (SR-F2) was effectively suppressed by the presence of scavenger receptor ligands^61^. By analogy, we speculate that, following axotomy, CED-1 on the surface of engulfing cells is initially functioning as a scavenger receptor and occupied by eat-me signals released from axon debris, which effectively block the adhesion function of CED-1. As axon debris is gradually removed by engulfing cells, CED-1 would switch its function from a scavenger receptor for debris removal to an adhesion molecule for neuronal regeneration. It remains to be seen whether eat-me signals released from axon debris inhibit the adhesion function of CED-1.

Recessive mutations in MEGF10 (SR-F3) in humans result in early-onset myopathy, areflexia, respiratory distress, and dysphagia (EMARDD), but the mechanism underlying the pathogenesis of the disease is unknown^62^. It was recently shown that the MEGF10 (SR-F3) mutation in mice leads to impaired proliferation and migration of myoblasts, which may contribute to the pathogenic process of EMARDD^62^. Here, our studies identify CED-1 in muscle cells as an adhesion molecule that promotes muscle protrusions and their interactions with regenerating axons, and have important implications for understanding mechanisms underlying neural circuit repair after injury or in neurodegenerative diseases.

## Methods

### Genetics and strain construction

*C. elegans* strains were cultured using standard methods^63^. All strains were grown at 20 °C. Either the *zdIs5[Pmec-4::GFP* transgene integrated into chromosome I*]* or the *zdIs4[Pmec-4::GFP* transgene integrated into chromosome IV*]* axonal marker was used in engulfment gene mutants for axon regeneration and axon debris study. The *zdIs5* marker strain has a higher regeneration baseline than the *zdIs4* marker strain. *zdIs4* (on chromosome IV) was introduced to *ced-1* (on chromosome I) mutants and *zdIs5* (on chromosome I) was introduced to *ced-5* (on chromosome IV) mutants to avoid unwanted secondary mutations generated during the strain construction due to chromosome recombination between adjacent loci of the marker insertion and the mutation. For easy comparison, mutants showing reduced regeneration were clustered together and results were compared in the *zdIs5* background whereas mutants showing enhanced regeneration were clustered together and results were compared in the *zdIs4* background as shown in Supplementary Figure 6. All mutants were analyzed in both *zdIs5* and *zdIs4* markers and mutant effects were comparable. A strain list appears as Supplementary Table 1.

### Transgenic animals

Germline transformation of *C. elegans* was performed using standard techniques^64^. For example, the *Punc-54::ced-1::GFP* transgene was injected at 35 ng/μl along with the coinjection marker *Podr-1::rfp* at 50 ng/μl. Transgenic lines were maintained by following *Podr-1::rfp* fluorescence.

### Plasmid construction

Standard protocol was used for the following plasmid constructions.

*Pced-1::myr::mCherry* The 5-kb *ced-1* promoter was amplified by PCR from genomic DNA. The PCR fragment was cloned into the PSM*::myr::mCherry* vector via FseI and AscI enzyme sites.

*Punc-54::GFP* The 1-kb *unc-54* promoter was amplified by PCR from genomic DNA, and was cloned into the PSM::*gfp* vector via FseI and AscI enzyme sites.

*Pmec-4::ced-1* The 3.3-kb *ced-1* cDNA was amplified by RT-PCR from total RNA and cloned into the *Pmec-4*::PSM vector via NheI and Asp718 enzyme sites.

*Punc-54::ced-1(N962A)* The N962A point mutation was introduced into the *ced-1* cDNA by site-directed mutagenesis and the mutation was confirmed by sequencing. The mutant *ced-1* cDNA *[ced-1(N962)]* was cloned into the *Punc-54*::PSM vector via NheI and Asp718 enzyme sites.

*Punc-54::ced-1ΔC* The *ced-1* cDNA lacking the region encoding the cytoplasmic domain (aa 1–930) was amplified by RT-PCR from total RNA and cloned into the *Punc-54*::PSM vector via NheI and Asp718 enzyme sites.

*Punc-54::slt-1 sp::ced-1 ecto::gfp* The endogenous *ced-1* signal peptide was replaced by the *slt-1* signal peptide. The *slt-1* signal peptide was fused to the *ced-1* ectodomain (aa 20–887) by PCR. The PCR fragment was cloned into the *Punc-54*::PSM::*gfp* vector via NheI and Asp718 enzyme sites.

*Pced-5::GFP* The 1.3-kb *ced-5* promoter was amplified by PCR from genomic DNA and cloned into the PSM::*gfp* vector via FseI and AscI enzyme sites.

*Plim-7::ced-5* The 4.1-kb *lim-7* promoter was amplified by PCR from genomic DNA, and was cloned into the PSM vector via FseI and AscI enzyme sites. The 8-kb *ced-5* genomic fragment was cloned into the *Plim-7*::PSM vector via BamHI and PspOMI enzyme sites.

*Pmec-4::ced-5* The 8-kb *ced-5* genomic fragment was cloned into the *Pmec-4::*PSM vector via BamHI and PspOMI enzyme sites.

*Punc-54::ced-6::gfp* The 1.5-kb *ced-6* cDNA was amplified by RT-PCR from total RNA. The *ced-6* cDNA was then cloned in frame into the *Punc-54::*PSM::*gfp* vector via AscI and Asp718 enzyme sites.

### Cell-type-specific RNAi

Cell-type-specific RNAi has been used in *C. elegans* to knockdown gene function in neurons^65^. In this study, we used the RNAi construct that contains inverted repeats separated by a linker sequence, from which hairpin-loop dsRNA is produced. The transgene containing the RNAi construct is expressed from a cell-type-specific promoter (*mec-4* for ALM neurons and *unc-54* for muscles).

### Laser axotomy

The device we used for femtosecond laser surgery is a cavity-dumped Ti:sapphire laser oscillator (Cascade Laser, KMLabs Inc., Boulder, CO) to generate laser pulses ~100 fs in duration and 200 kHz in repetition rate. The laser pulses were tightly-focused onto targeted axons using a Nikon ×100, 1.4 NA oil-immersion objective. The vaporization threshold corresponds to pulse energies of 5–15 nJ. Successful laser axotomy was confirmed by visualizing the targeted area immediately after surgery.

### Monitoring and quantifying axonal debris and regeneration

The morphology of neuronal cell bodies, axon debris, regenerating axons, and muscles was based on high-magnification Z-stacks using a Zeiss ×60, 1.4 NA oil-immersion objective. We mounted individual animals on 2% agar pads and anaesthetized them with 3 mM sodium azide, the lowest possible concentration to keep adult animals immobilized. Laser axotomy was performed and worms were recovered within 10 minutes of sodium azide treatment. Recovered worms were placed on fresh plates with bacterial foods and imaged 2.5–12 h (Fig. 1c) or 24 h after axotomy using a Hamamatsu ORCA AG camera.

The axon length of regenerating neurons was quantified 24 h after surgery. Axon lengths were calculated as the actual contour length between the injury site and axon termini measured along the cylindrical surface of each worm, by tracing the axon through a 3-dimensional image stack. *P* values for the length measurements were calculated using a Student’s *t*-Test. All experiments were carried out in duplicate but only one result was shown.

### Statistics

Average data of axon length are presented as means ± SEM. Data of % animals retaining axon debris, % ALM axons adhered to muscles, % ALM axons with filopodia, and % muscle contacts with enriched CED-1::mRFP fusion proteins are presented as proportions ± SEP. Statistical analyses were carried out with the Primer of Biostatistics software for the Student’s *t*-Test and the two-proportion *Z*-test. *P* < 0.05 was considered statistically significant and *P* < 0.01 or *P* < 0.001 was considered statistically very significant.

### Reporting summary

Further information on experimental design is available in the Nature Research Reporting Summary linked to this paper.

## Electronic supplementary material

Supplementary Information

Reporting Summary

## Data Availability

The authors declare that all data supporting the findings of this study are available within the paper and its supplementary information file.
