# Supplementary material for: Engulfing cells promote neuronal regeneration and remove neuronal debris through distinct biochemical functions of CED-1
Source: Nat Commun. 2018 Nov 19;9:4842. doi: 10.1038/s41467-018-07291-x (PMC6242819; doi:10.1038/s41467-018-07291-x)
Supplement: Supplementary file 1 — Supplementary Information [file 41467_2018_7291_MOESM1_ESM.pdf]

**Supplementary information**

# Engulfing cells promote neuronal regeneration and remove neuronal debris through distinct biochemical functions of CED-1

Chiu et al.

**Supplementary Figure Legends**

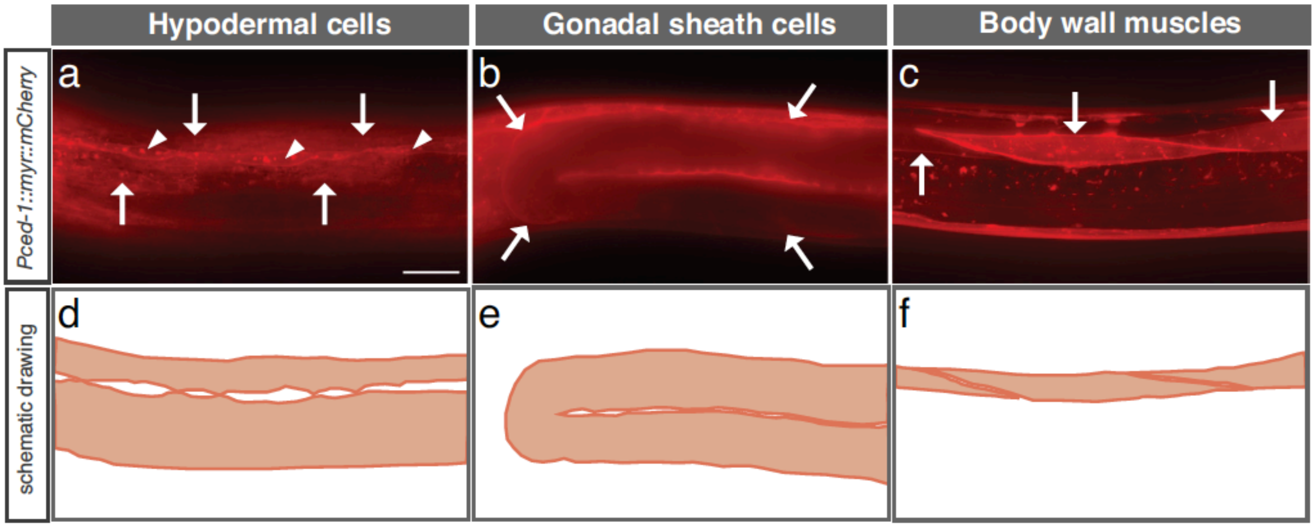


**Supplementary Figure 1 | The expression pattern of a 5-kb *ced-1* promoter reporter at the adult stage.** (a) Arrows point to the expression of the *Pced-1::myr::mCherry* reporter in syncytial hypodermal cells, which envelop the main body of worms. Seam cells, indicated by arrowheads, locate at the lateral sides of the worm body. (b) The expression of the *Pced-1::myr::mCherry* reporter was observed in gonadal sheath cells. Arrows point to the U-shape gonad in the anterior part of the hermaphrodite. (c) Body wall muscles, spindle-shape cells indicated by arrows, expressed the *Pced-1::myr::mCherry* reporter at the adult stage. The expression level of CED-1 is not significantly changed after injury. (d-f) The schematic drawing corresponds to each type of engulfing cells. Anterior is to the left; dorsal is up in all panels. Scale bar: 20 μm.

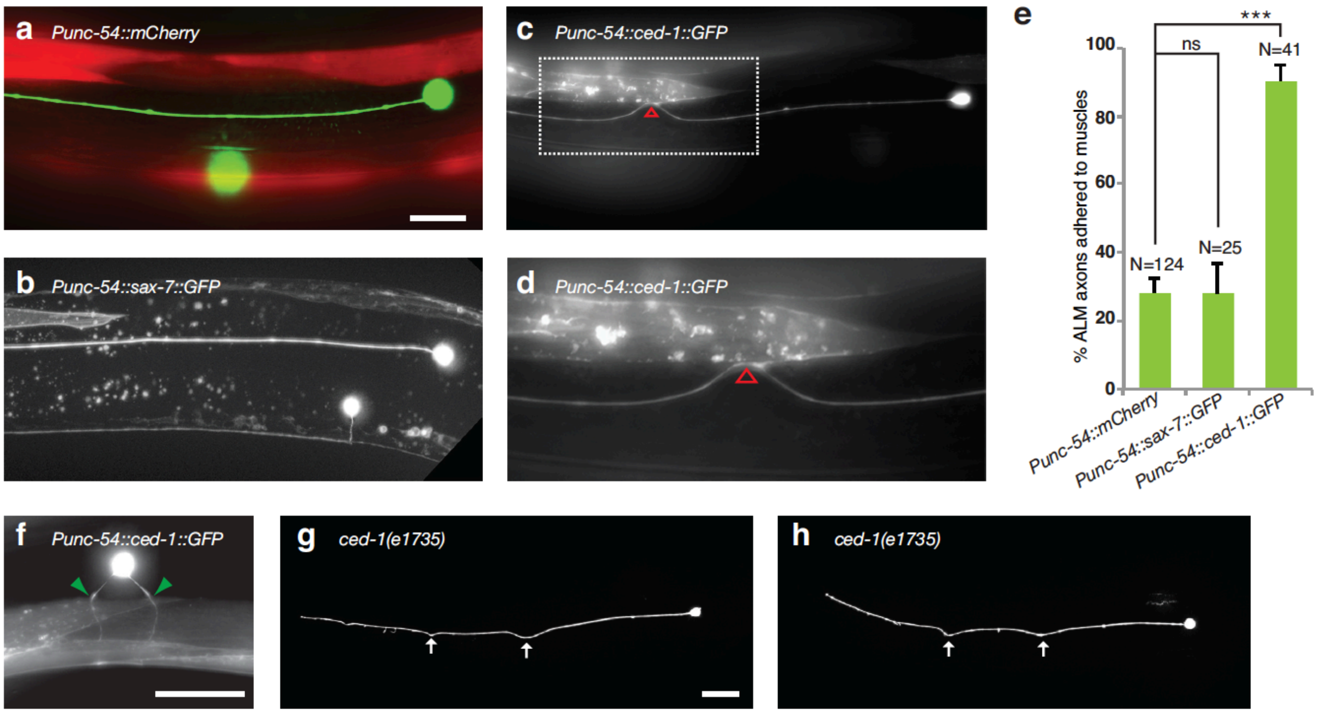


**Supplementary Figure 2 | ALM and AVM axons were adhered to body wall muscles expressing the CED-1 transmembrane protein.** (a-d) Representative images showing ALM axons and body wall muscles. Images were taken in L4 stage for initial axon trajectory. Anterior is to the left; dorsal is up. Scale bar: 20 μm. (a, b) ALM axons in transgenic animals expressing the *Punc-54::mCherry* (a) or the *Punc-54::sax-7* (b) transgene were not adhered to body wall muscles. (c, d) ALM axons in transgenic animals expressing the *Punc-54::ced-1* transgene were adhered to body wall muscles. The dashed box area in S2**c** was blown up and shown in S2**d**. Red open arrowheads in S2**c** and S2**d** point to axon contact points. (e) The percentages of ALM axons adhered to body wall muscles in development. The N number represents the number of animals analyzed. Error bars represent SEP. Asterisks represent P<0.001 by *Z*-test for two proportions. (f) AVM axons in the transgenic animal expressing the *Punc-54::ced-1* transgene were adhered to

body wall muscles. Green arrowheads point to axon contact points. Scale bar: 20 μm. (g, h) ALM axons in *ced-1(e1735)* mutants showed curved trajectory. Arrows mark sharp turns in ALM axons. Scale bar: 20 μm.

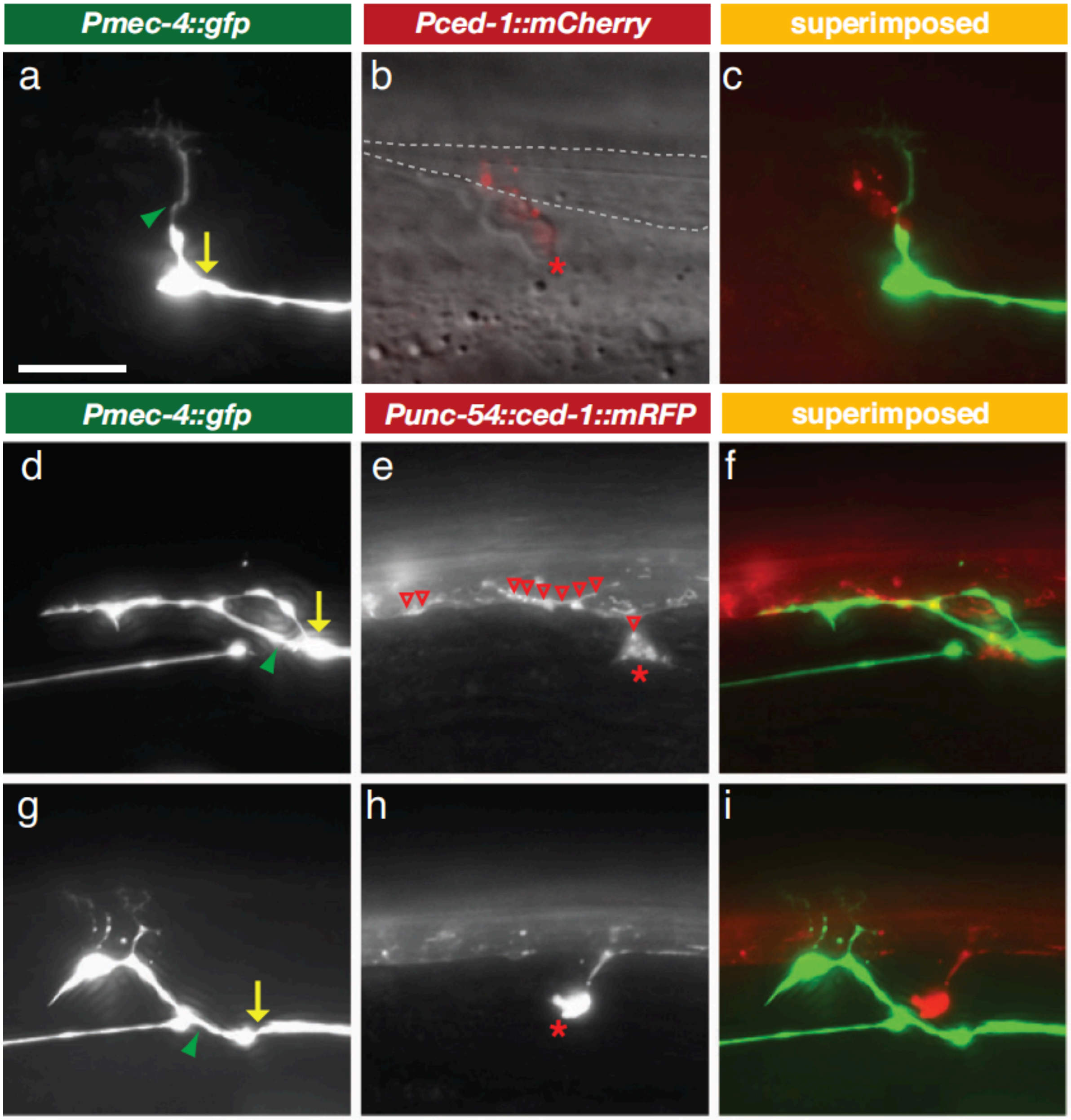


**Supplementary Figure 3 | *In vivo* interactions between body wall muscles and regenerating ALM axons.** (a-i) Representative images showing interactions between body wall muscles and regenerating axons. (c, f, i) Merged images. Regenerating axons were labeled by the *Pmec-4::GFP* transgene (a, d, g) and body wall muscles were labeled by the *Pced-1::mCherry* (b) or the *Punc-54::ced-1::mRFP* reporter (e, h). Yellow arrows,

green arrowheads, and asterisks point to lesion sites, regenerating axons, and muscle protrusions, respectively. Dashed lines in S3b outlined the border of a body wall muscle cell. Red open arrowheads in S3e indicate enrichment of the CED-1::mRFP fusion protein in muscles at regions contacting with regenerating axons. Anterior is to the left; dorsal is up in all panels. Scale bar: 10 μm.

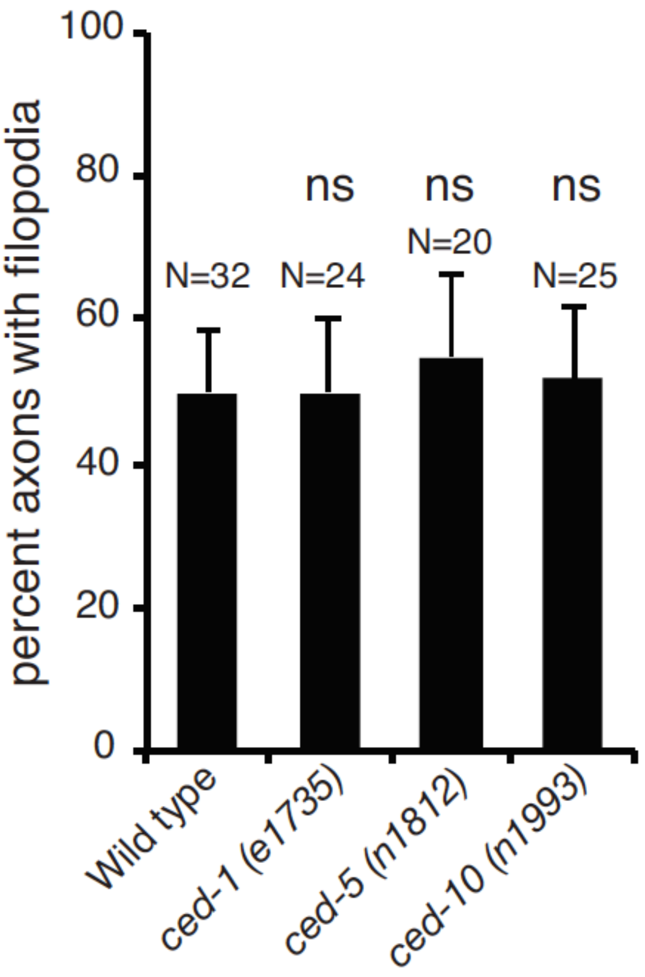


**Supplementary Figure 4 | *ced-1*, *ced-5*, and *ced-10* mutations do not affect initial filopodial formation.** The percentages of severed ALM axons displaying filopodia 10 hours after injury. Error bars represent SEP. ns indicates no significant difference by Z-test for two proportions.

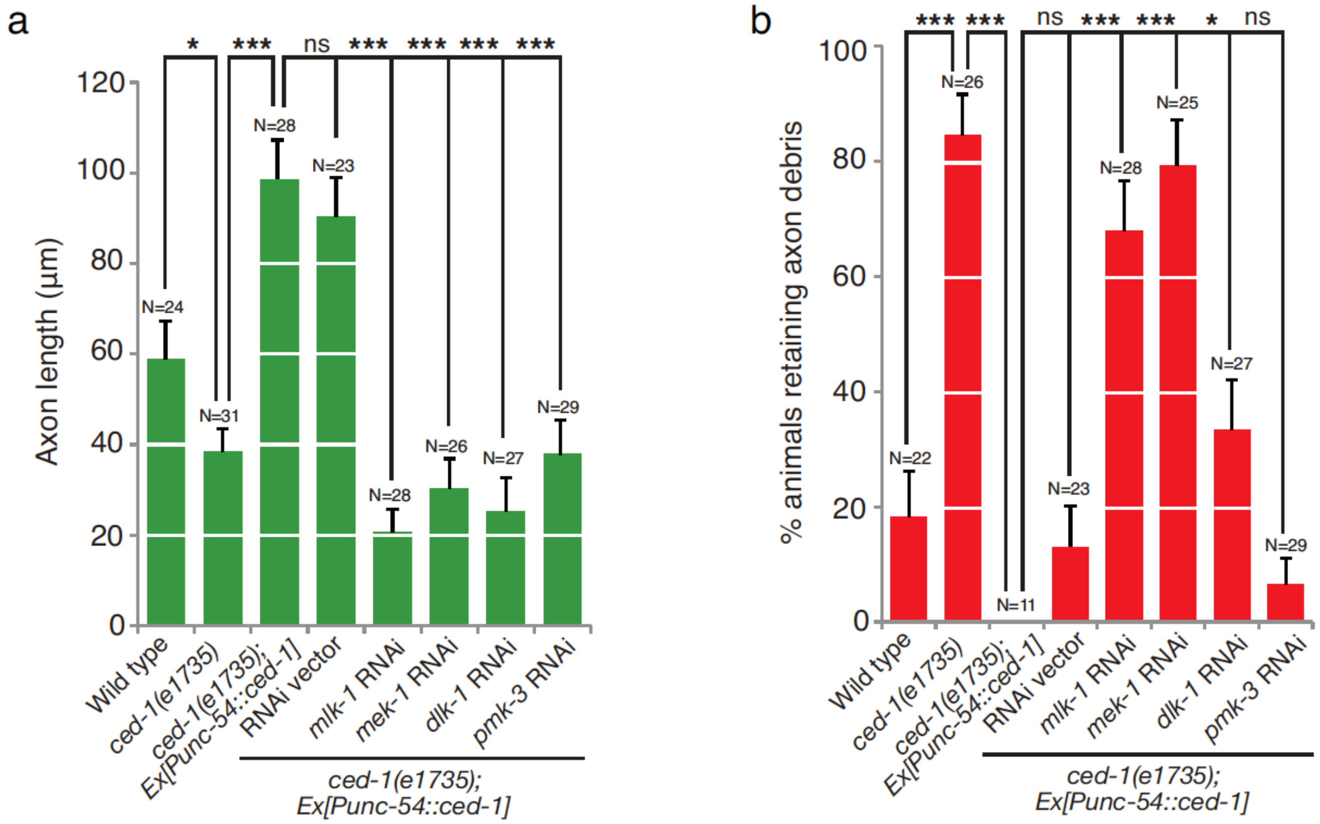


**Supplementary Figure 5 | The role of p38 and JNK signaling pathways in CED-1-mediated axon regeneration and axon debris clearance.** (a) Average length of regenerating ALM axons 24 hours after laser axotomy. Axons were visualized using the *zdIs4[Pmec-4::GFP]* marker. ns indicates no significant difference. Error bars indicate SEM. * and *** represent $p < 0.05$ and $p < 0.001$, respectively. P values were calculated using a Student's t-Test. RNAi vector control did not significantly affect CED-1-mediated axon regeneration. (b) The percentages of animals retaining axon debris 24 hours after laser axotomy. ns indicates no significant difference. Error bars represent SEP. * and *** denote $p < 0.05$ and $p < 0.001$, respectively, by Z-test for two proportions. RNAi vector control did not significantly affect CED-1-mediated axon debris clearance.

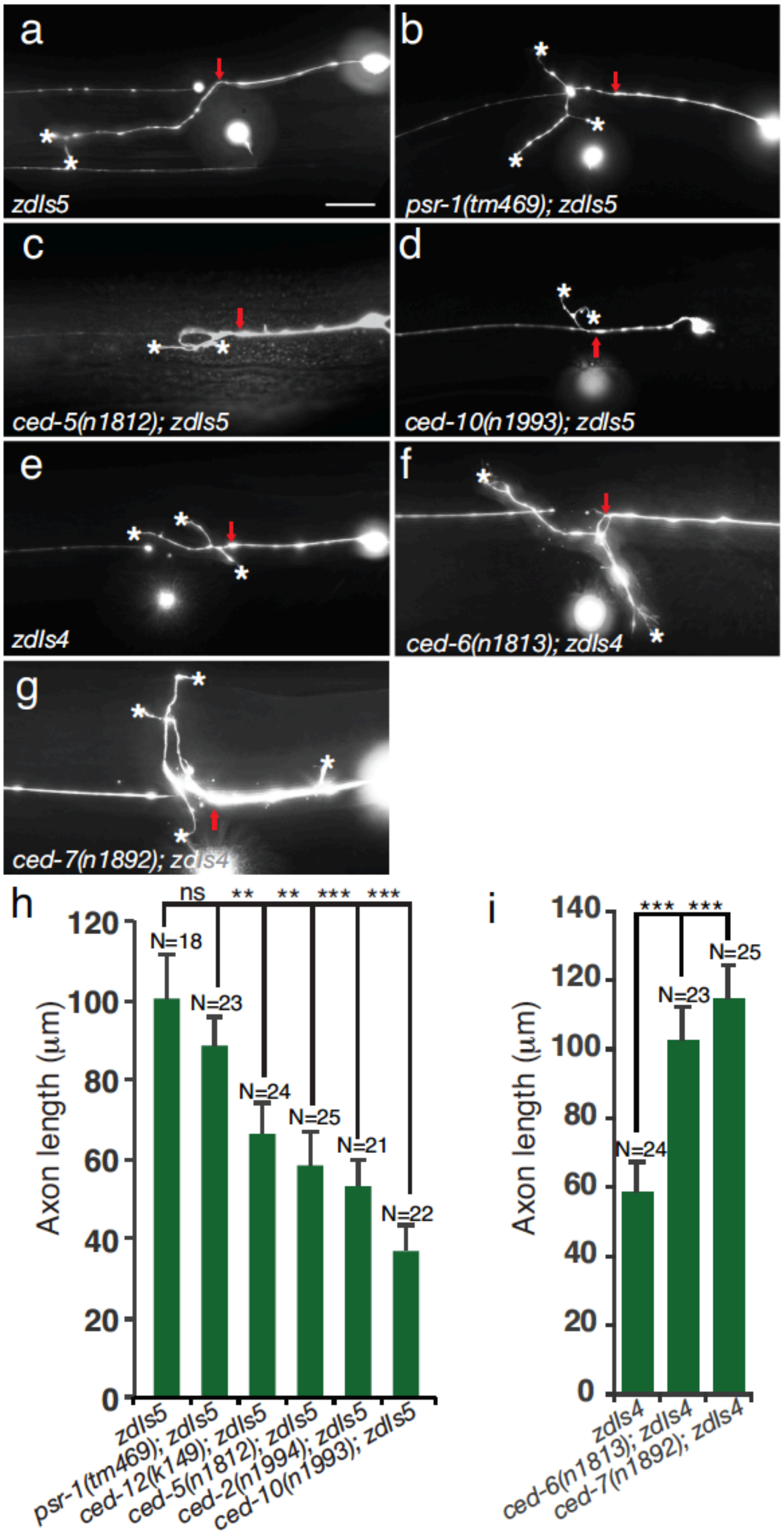

a
zdIs5
b
psr-1(tm469); zdIs5
c
ced-5(n1812); zdIs5
d
ced-10(n1993); zdIs5
e
zdIs4
f
ced-6(n1813); zdIs4
g
ced-7(n1892); zdIs4
h
Axon length (μm)
ns
**
**
***
***
N=18
N=23
N=24
N=25
N=21
N=22
zdIs5
psr-1(tm469); zdIs5
ced-12(k149); zdIs5
ced-5(n1812); zdIs5
ced-2(n1994); zdIs5
ced-10(n1993); zdIs5
i
Axon length (μm)
***
***
N=24
N=23
N=25
zdIs4
ced-6(n1813); zdIs4
ced-7(n1892); zdIs4

**Supplementary Figure 6 | Selective engulfment genes are regulators of axon regeneration.** (a-g) Representative examples showing the capacity of wild-type animals as well as different engulfment gene mutants to regenerate axons after laser axotomy. Axon regeneration was analyzed in either *zdIs5* (a-d) or *zdIs4* (e-g) strains, which carry an axonal marker *[Pmec-4::GFP]* integrated into different chromosomes, dependent on the ease of strain construction. Arrows indicate lesion sites. Asterisks point to termini of regenerating axons. Anterior is to the left; dorsal is up in all images. Scale bar: 20 μm. (a) Wild-type animals (*zdIs5*) regenerated axons robustly. (b) The regeneration ability of *psr-1(tn469)* mutants is similar to that of wild-type animals. (c) *ced-5(n1812)* mutants showed limited axon regeneration. (d) *ced-10(n1993)* mutants displayed reduced axon regeneration. (e) Wild-type animals (*zdIs4*) regrew axons considerably. (f) *ced-6(n1813)* mutants regenerated longer axons. (g) *ced-7(n1892)* mutants displayed enhanced axon regeneration. (h, i) Average length of regenerating ALM axons. The N number represents the number of animals analyzed. ns indicates no significant difference. Error bars indicate SEM. ** and *** denote $p < 0.01$ and $p < 0.001$, respectively. P values were calculated using a Student's t-Test.

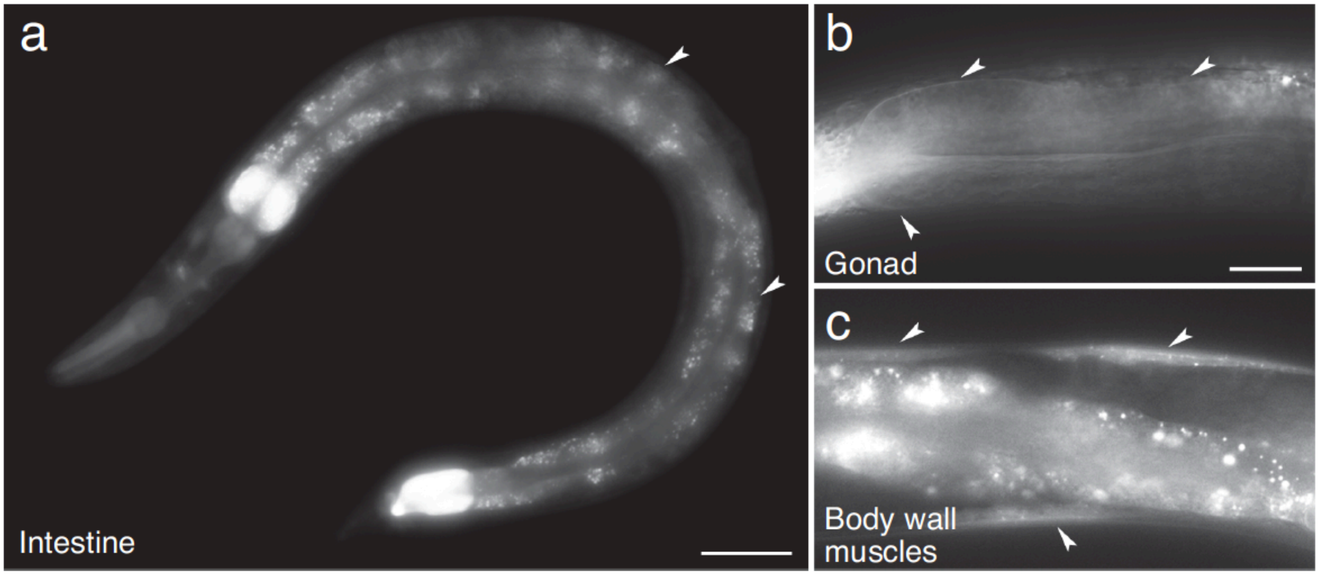


**Supplementary Figure 7 | The expression pattern of a 1.3-kb *ced-5* promoter::GFP reporter at the adult stage.** (a-c) The expression of the *Pced-5::GFP* reporter at the adult stage was detected in various cells indicated by arrowheads in each image, including intestinal cells (a), the gonad (b), and body wall muscles (c). Anterior is to the left; dorsal is up. Scale bar: 100 μm in (a) and 20 μm in (b, c).

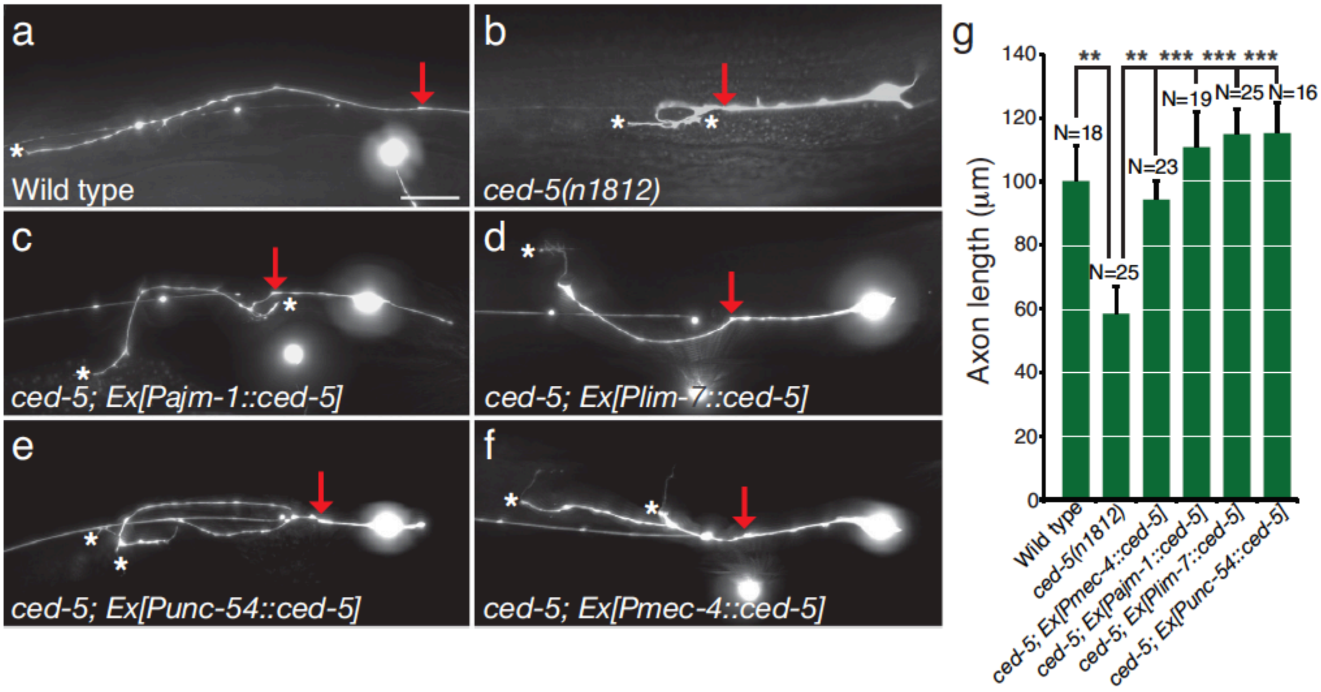


**Supplementary Figure 8 | *ced-5* functions in touch neurons and three types of engulfing cells to promote ALM axon regeneration.** (a-f) Representative images showing patterns of axon regeneration in wild-type animals, *ced-5* mutants, and *ced-5* mutants carrying different transgenes. Axon trajectories were visualized using the *zdIs5[Pmec-4::GFP]* marker. Anterior is to the left; dorsal is up in all images. Scale bar: 20 μm. (a) Wild-type animals were capable of robust regeneration 24 hours after laser axotomy. (b) *ced-5(n1812)* mutants exhibited reduced axon regeneration. (c-f) Axon regeneration in *ced-5(n1812)* mutants carrying various transgenes. Reduced axon regeneration was rescued in *ced-5* mutants carrying the *Pajm-1::ced-5* transgene (c), the *Plim-7::ced-5* transgene (d), the *Punc-54::ced-5* transgene (e), or the *Pmec-4::ced-5* transgene (f). (g) Average length of regenerating ALM axons. The N number represents the number of animals analyzed. Error bars indicate SEM. ** and *** represent $p < 0.01$ and $p < 0.001$, respectively. P values were calculated using a Student's t-Test.

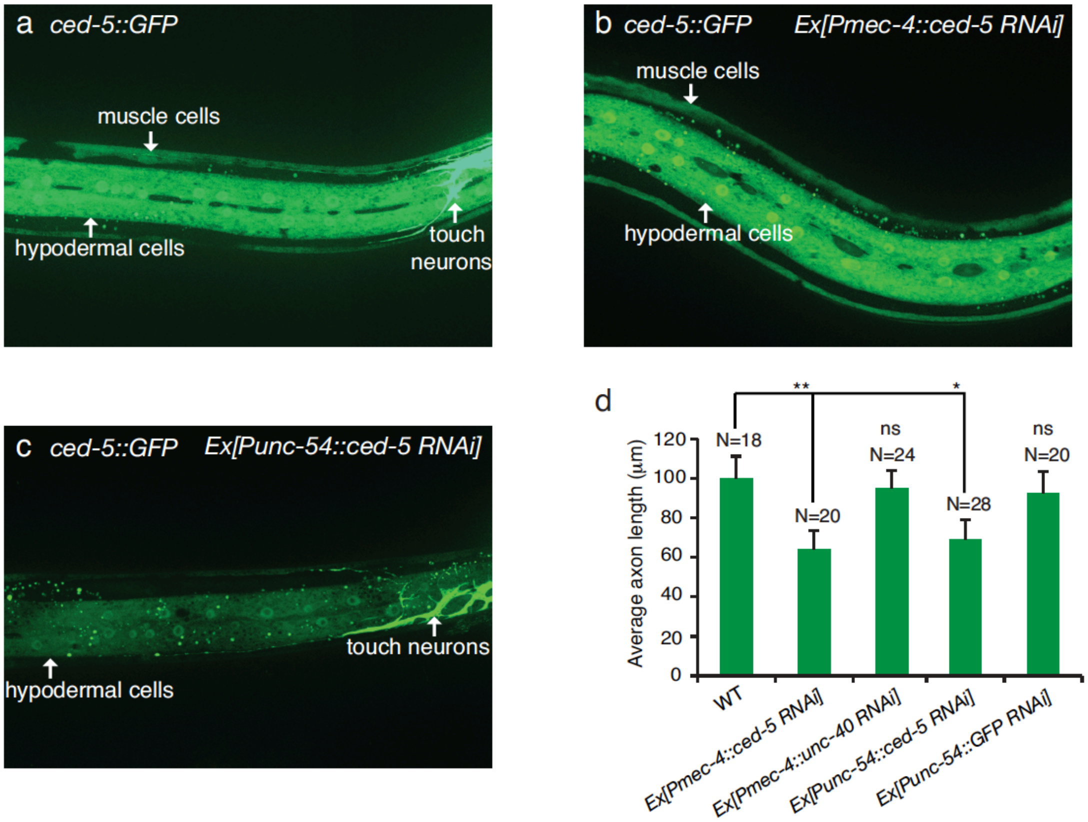


**Supplementary Figure 9 | Reduction of *ced-5*'s functions in touch neurons or muscle cells reduced ALM axon regeneration.** (a) The expression of the CED-5::GFP fusion protein in touch neurons, body wall muscle cells, and hypodermal cells. (b) *ced-5* RNAi in touch neurons preferentially silenced the expression of the CED-5::GFP fusion protein in touch neurons. (c) *ced-5* RNAi in muscle cells preferentially silenced the expression of the CED-5::GFP fusion protein in muscle cells. (d) Average length of regenerating ALM axons 24 hours after injury in wild type and transgenic animals expressing the *ced-5* RNAi transgenes (or the control RNAi transgenes) from the *mec-4* (touch neurons specific) or the *unc-54* (muscles specific) promoter. Error bars indicate SEM. ** and * indicate $p < 0.01$ and $p < 0.05$, respectively. P values were calculated using a Student's t-Test.

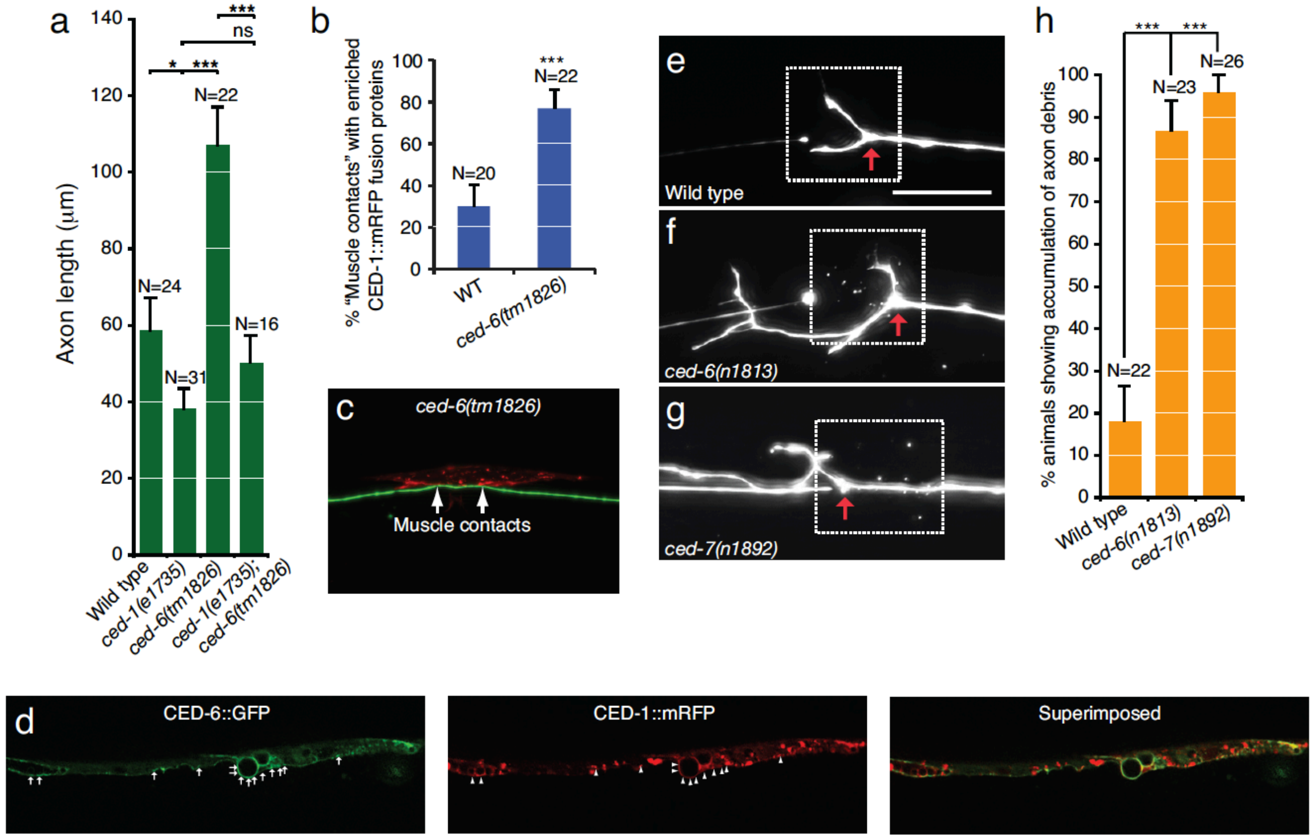


**Supplementary Figure 10 | The genetic interaction between *ced-1* and *ced-6* genes in ALM axon regeneration.** (a) Average length of regenerating ALM axons 24 hours following axotomy. Axon trajectories were visualized using the *zdIs4[Pmec-4::GFP]* marker. ns indicates no significant difference. Error bars indicate SEM. * and *** represent $p < 0.05$ and $p < 0.001$, respectively. P values were calculated using a Student's t-Test. (b) Frequency of enriched CED-1::mRFP fusion proteins on the muscle surface in wild type versus *ced-6* mutants. Error bars represent SEP. (c) A representative image showing enriched CED-1::mRFP fusion proteins at muscle contacts. (d) Distribution of CED-6::GFP and CED-1::mRFP fusion proteins in muscle cells. Arrows indicate CED-6::GFP fusion proteins whereas arrowheads mark CED-1::mRFP fusion proteins co-

localization to the surface area of muscle cells. (e-h) Axon regenerated well in spite of defects in axon debris removal in *ced-6* and *ced-7* mutants. Representative images showing regenerating ALM axons in wild-type animals (e), *ced-6* mutants (f), and *ced-7* mutants (g) 24 hours after axotomy. (f, g) Accumulated axon debris can be seen in *ced-6* and *ced-7* mutants. Axon trajectories and debris were visualized using the *zdIs4[Pmec-4::GFP]* marker. Scale bar: 20 μm. (h) The percentages of animals accumulating axon debris 24 hours after laser axotomy. Error bars represent SEP. *** denotes $p < 0.001$ by Z-test for two proportions.

## Supplementary Table 1 A strain list

| Strains | Mutations | Integrated transgenes | Extrachromosomal transgenes |
|---|---|---|---|
| SK4005 | | *zdIs5 [Pmec-4::gfp] I* | |
| XN430 | | *zdIs5 I* | *xrEx210[Pced-1::mCherry; Podr-1::rfp]* |
| XN810 | | *zdIs5 I* | *xrEx289[Punc-54::mCherry]* |
| CX7609 | | *zdIs4 [Pmec-4::gfp] IV* | |
| XN11 | *ced-1(e1735) I* | *zdIs4 IV* | |
| XN705 | *ced-1(e1735) I* | *zdIs4 IV* | *xrEx261[Pmec-4::ced-1; Podr-1::rfp]* |
| XN713 | *ced-1(e1735) I* | *zdIs4 IV* | *xrEx263[Pajm-1::ced-1::gfp; Podr-1::rfp]* |
| XN801 | *ced-1(e1735) I* | *zdIs4 IV; bcIs39[Plim-7::ced-1::gfp; lin-15(+)] V* | |
| XN1197 | *ced-1(e1735) I* | *zdIs4 IV* | *xrEx303[Punc-54::ced-1::gfp; Podr-1::rfp]* |
| XN1109 | *ced-1(e1735) I* | *zdIs4 IV* | *xrEx398[Punc-54::ced-1(N962A)::gfp; Podr-1::rfp]* |
| XN1192 | *ced-1(e1735) I* | *zdIs4 IV* | *xrEx412[Punc-54::ced-1ΔC::gfp; Podr-1::rfp]* |
| XN1416 | *ced-1(e1735) I* | *zdIs4 IV* | *xrEx523[Punc-54::slt-1 sp::ced-1 ecto::gfp; Podr-1::rfp]* |
| XN898 | *ced-1(e1735) I* | *zdIs4 IV* | *xrEx310[Punc-54::ced-5; Podr-1::rfp]* |
| XN1325 | *ced-1(e1735) I; ced-5(n1812) IV* | *zdIs4 IV* | *xrEx303* |
| XN1094 | *ced-1(e1735) I; ced-6(tm1826) III* | *zdIs4 IV* | *xrEx303* |
| XN1800 | *ced-1(e1735) I; ced-6(tm1826) III* | *zdIs4 IV* | |
| XN946 | | | *xrEx340[Pced-5::gfp; Podr-1::rfp]* |
| XN736 | *ced-5(n1812) IV* | *zdIs5 I* | |
| XN812 | *ced-5(n1812) IV* | *zdIs5 I* | *xrEx291[Pmec-4::ced-5; Podr-1::rfp]* |
| XN885 | *ced-5(n1812) IV* | *zdIs5 I* | *xrEx304[Pajm-1::ced-5; Podr-1::rfp]* |
| XN887 | *ced-5(n1812) IV* | *zdIs5 I* | *xrEx306[Plim-7::ced-5; Podr-1::rfp]* |
| XN830 | *ced-5(n1812) IV* | *zdIs5 I* | *xrEx297[Punc-54::ced-5; Podr-1::rfp]* |
| XN1064 | *ced-6(n1813) III* | *zdIs4 IV* | |
| XN1111 | *ced-6(tm1826) III* | *zdIs4 IV* | *xrEx378[Punc-54::ced-1::mRFP; Podr-1::rfp]* |
| XN1113 | *ced-7(n1892)* | *zdIs4 IV* | |
| XN912 | *psr-1(tm469) IV* | *zdIs5 I* | |
| CX5683 | *ced-10(n1993) IV* | *zdIs5 I* | |
| XN889 | *ced-2(n1994) IV* | *zdIs5 I* | |
| XN1178 | *ced-12(k149) I* | *zdIs5 I* | |
| CX5300 | *unc-6(ev400) X* | *zdIs5 I* | |
| XN1661 | *unc-6(ev400) X* | *zdIs5 I* | *xrEx303* |
| XN1467 | | *xrIs37[PF49H12.4::gfp; Pmec-3::mCherry] IV* | |
| XN1591 | *sax-7(xr35)* | *xrIs37 IV* | |
| XN2152 | *sax-7(xr35)* | *xrIs37 IV* | *xrEx797[Punc-54::ced-1::gfp; Podr-1::rfp]* |
| XN2285 | | | *xrEx303; xrEx820[Punc-54::TagRFP::rab-5; Podr-1::gfp]* |
| XN2286 | | | *xrEx303; xrEx821[Punc-54::TagRFP::rab-7; Podr-1::gfp]* |
| XN1959 | | | *xrEx469[Punc-54::slt-1 sp::ced-1 ecto::gfp; Podr-1::rfp]; xrEx685[Pmec-4::mCherry; egl-20::gfp]* |
| XN1281 | | *xrIs33[Punc-54::ced-1::mRFP; Podr-1::rfp]* | *xrEx400[Punc-54::ced-6::gfp; Podr-1::rfp]* |
